# Characterization of the Three New Kayviruses and Their Lytic Activity Against Multidrug-Resistant *Staphylococcus aureus*

**DOI:** 10.3390/microorganisms7100471

**Published:** 2019-10-18

**Authors:** Natalia Łubowska, Bartłomiej Grygorcewicz, Katarzyna Kosznik-Kwaśnicka, Agata Zauszkiewicz-Pawlak, Alicja Węgrzyn, Barbara Dołęgowska, Lidia Piechowicz

**Affiliations:** 1 Department of Medical Microbiology, Faculty of Medicine, Medical University of Gdańsk, 80-204 Gdańsk, Poland; nlubowska@gumed.edu.pl; 2 Department of Laboratory Medicine, Chair of Microbiology, Immunology and Laboratory Medicine, Pomeranian Medical University in Szczecin, 70-111 Szczecin, Poland; bartlomiej.grygorcewicz@pum.edu.pl (B.G.); barbara.dolegowska@pum.edu.pl (B.D.); 3 Laboratory of Molecular Biology, Institute of Biochemistry and Biophysics, Polish Academy of Sciences, 80-819 Gdańsk, Poland; katarzyna.kwasnicka@biol.ug.edu.pl (K.K.-K.); alicja.wegrzyn@biol.ug.edu.pl (A.W.); 4 Department of Histology, Faculty of Medicine, Medical University of Gdańsk, 80-211 Gdańsk, Poland; agata.zauszkiewicz-pawlak@gumed.edu.pl

**Keywords:** bacteriophage, *Staphylococcus aureus*, *Kayvirus*

## Abstract

The development of antimicrobial resistance has become a global concern. One approach to overcome the problem of drug resistance is the application of bacteriophages. This study aimed at characterizing three phages isolated from sewage, which show lytic activity against clinical isolates of multidrug-resistant *Staphylococcus aureus*. Morphology, genetics and biological properties, including host range, adsorption rate, latent time, phage burst size and lysis profiles, were studied in all three phages. As analyzed by transmission electron microscopy (TEM), phages vB_SauM-A, vB_SauM-C, vB_SauM-D have a myovirion morphology. One of the tested phages, vB_SauM-A, has relatively rapid adsorption (86% in 17.5 min), short latent period (25 min) and extremely large burst size (~500 plaque-forming units (PFU) per infected cell). The genomic analysis revealed that vB_SauM-A, vB_SauM-C, vB_SauM-D possess large genomes (vB_SauM-A 139,031 bp, vB_SauM-C 140,086 bp, vB_SauM-D 139,088 bp) with low G+C content (~30.4%) and are very closely related to the phage K (95–97% similarity). The isolated bacteriophages demonstrate broad host range against MDR *S. aureus* strains, high lytic activity corresponding to strictly virulent life cycle, suggesting their potential to treat *S. aureus* infections.

## 1. Introduction

*Staphylococcus aureus* is the prime cause of hospital-acquired infections. It is associated with a number of diseases in humans, ranging from superficial to invasive infections such as pneumonia and sepsis [1]. *S. aureus* infections can be difficult to treat because of the prevalence of *S. aureus* strains resistant to β-lactam antibiotics (methicillin-resistant *Staphylococcus aureus*—MRSA). In 2017, the percentage of invasive MRSA isolates in Europe ranged from 1% in Norway to 44.4% in Romania, with a population-weighted mean of 16.9% [2]. Treatment of *S. aureus* infections is a challenge not only because of MRSA resistance towards β-lactams antibiotics but because many isolates exhibit resistance to other antimicrobial agents, for example, macrolides, tetracycline, aminoglycosides, chloramphenicol and fluoroquinolones, leading to the emergence of multidrug resistant *S. aureus* (MDRSA). The rate at which *S. aureus* can develop or acquire resistance to new antibiotics, is actually higher than that, at which new antibiotics are discovered and developed [3]. Problems connected with antibiotic treatment necessitate the investigation and development of novel treatment strategies.

A potential solution is the use of bacteriophages–viruses that specifically lyse bacterial cell. Their host specificity ranges from a few strains of a bacterial species to, more rarely, more than one relatively closely related bacterial genus. Thus, they do not destroy beneficial bacterial flora and the risk of developing secondary infections is minimalized [4,5]. Owing to relatively narrow host range of most phages, the number of bacterial types with which the selection for specific phage-resistance mechanisms can occur, is limited [4]. Moreover, spontaneous mutations may confer phage–bacteria coevolution, therefore phages can overcome bacterial resistance [6]. Although phage specificity is an advantage in the case of the minimal impact of natural flora, it can be also seen as a problem in therapy. Because phages exhibit such a narrow host range, there is a need to identify the etiologic agent and select a phage lytic for the targeted bacterial strain before using phages therapeutically. This involves creating an extensive collection of well-characterized phage for a broad range of pathogens [7]. Kayviruses are polyvalent bacteriophages infecting a broad spectrum of *S. aureus* and other staphylococci. These bacteriophages are ones of the best agents that can be used to treat staphylococcal infection [8,9,10,11]. Therefore, some of them are used in commercial bacteriophage preparations [8,10,12].

The aim of this work was to isolate and characterize *S. aureus* bacteriophages in a view of their potential application in phage therapy.

## 2. Materials and Methods

### 2.1. Bacterial Strains

A total of 88 bacterial strains, including 21 representatives of different species (Table 2) and 67 *S. aureus* clinical isolates (Table 3) from different clinical materials (wound, pus, furuncle, bronchial tree), of both inpatients (*n* = 56) and outpatients (*n* = 11) were used in the study. The isolates originated from the collection of the Department of Medical Microbiology, Medical University of Gdansk and were obtained from 7 clinical centers of Gdansk region between 2013 and 2015. All isolates were identified by conventional bacteriological tests and antimicrobial susceptibility testing was performed by means of the disk diffusion method according to the guidelines of Clinical and Laboratory Standards Institute. All the strains tested were confirmed to be methicillin-resistant. Moreover, the strains were also resistant to erythromycin (96%), clindamycin (91%), norfloxacin (76%) and ciprofloxacin (76%), sulfamethoxazole/trimethoprim (7%), tetracycline (33%), chloramphenicol (27%), gentamicin (10%). All strains assayed were susceptible to fusidic acid, mupirocin and linezolid. Bacteria were stored at −80°C in tryptic soy broth (TSB) supplemented with 10% glycerol. All strains were cultured in Luria-Bertani broth (LB; Biomaxima, Lublin, Poland) or on LB agar plates (LA) solidified with 1.5% (*w*/*v*) agar (Becton Dickinson, Franklin Lakes, NJ, USA).

### 2.2. Isolation and Purification of Phages

Samples (*n* = 86) collected from wastewater treatment plants were used for isolation of bacteriophages according to Guzmán et al. [13]. Approximately 10 mL of sample was centrifuged at 2500 × g for 20 min and filtered through 0.2 μm Minisart^®^ filters (Sartorius Stedim Biotech, Goettingen, Germany). Sample was mixed with the culture of different strains of *Staphylococcus aureus* in its mid-exponential phase of growth (an optical density at 600 nm [OD_600 nm_] of 0.6), growing in 2 × TSB supplemented with 10 mM of MgSO_4_ and CaCl_2_. Samples were incubated with rotary shacking (180 rpm, at 37 °C). Chloroform was added to the culture following incubation and samples were placed on a rotor shaker for the next 15 min. Afterwards, tubes were centrifuged 2500 × g for 20 min and filtered through the 0.2 μm filter. Phage activity was tested by a double overlay agar plaque assay.

The clear plaque was picked and phages were suspended with SMG buffer (100 mM NaCl, 8 mM MgSO_4_, 50 mM Tris-HCl [pH 7.5], 0.01% gelatin). Isolated phages were purified by a triple transfer of a single plaque and propagation until homologous plaques were obtained. Decimal dilutions of lysates in SM buffer (100 mM NaCl, 8 mM MgSO_4_, 50 mM Tris-HCl [pH 7.5]) were prepared to define the titer of bacteriophage and subjected to plaque assay using the double agar layer method. Plaques were counted on the dilution plate in spots containing 30–300 plaques. Results were expressed in plaque forming units per mL (PFU/mL).

### 2.3. Phage Propagation

To amplify the phages, 5 mL of bacterial host strain grown overnight in liquid LB medium were added to 500 mL of LB and incubated at 37 °C with agitation. When the OD of the culture reached 0.1 at a 600 nm wavelength, the bacteria were infected with phages at a multiplicity of infection (MOI) of 0.1 and incubated at 37 °C until lysis occurred [14]. For phage purification, polyethylene glycol (PEG) 8000 (BioShop, Burlington, Ontario, Canada) was added (final concentration 10% *w*/*v*) and stirred overnight at 4 °C [15]. The precipitate was collected by centrifugation at 11,000× g for 20 min at 4 °C and suspended in TM buffer (10 mM Tris-HCl, 10 mM MgSO_4_ [pH 7.2]). PEG8000 was removed by adding the same volume of chloroform, vortexing and centrifugation at 3000× g for 15 min [16]. Phages for Transmission Electron Microscopy (TEM) were purified by centrifugation in CsCl gradients at 95,000× g for 2.5 h [17].

### 2.4. Electron Microscopy Imaging of Phages

Phage particles in buffer were deposited on Formvar/Carbon-coated copper grids and negatively stained with 1% uranyl acetate in water (pH 4) [18]. Visualization was performed using a transmission electron microscope (TEM) (JEM 1200EX II; Jeol Ltd., Akishima, Tokyo, Japan) operated at 100 kV. The size of the head and length of the tail were calculated from 5 independent measurements of separate virions and reported as a mean value ± standard deviation [19].

### 2.5. Efficiency of Phage Adsorption

An adsorption assay was performed according to the protocol described before, with some modifications [20]. Overnight liquid cultures of the bacterial strains were diluted 1:100 in LB medium and incubated at 37 °C until the OD_600_ reached 0.1 value. Samples of 2 mL were centrifuged at 2000× g for 5 min and pellets were suspended in 1.5 mL LB. After 15 min of incubation at 37 °C, the phages were added to each culture at a MOI of 0.01. Samples were withdrawn immediately after the phage addition (time zero) and at indicated time points and centrifuged at 8000× g for 30 s. Subsequently, the supernatants were titrated to determine the number of unabsorbed phages. The whole experiment was replicated thrice. A sample withdrawn immediately after addition of bacteriophages to the cell suspension was considered as 100% non-adsorbed phages.

### 2.6. One-Step Growth Analysis

Lytic development of phages was studied in one-step growth experiment, following the protocol described previously [20,21], with minor modifications. Host bacteria were grown in LB medium at 37 °C to OD_600_ = 0.1. Bacterial culture of 10 mL was centrifuged (2000× g for 5 min at 4 °C) and the pellet was suspended in 5 mL of LB medium with 3 mM sodium azide (Sigma Aldrich, Saint Louis, MO, USA). The sample was centrifuged again after 10 min of incubation at 37 °C and the pellet was resuspended in 5 mL of fresh LB medium. Following 10-min incubation at 37 °C, the phage was added to MOI of 0.05 and allowed to adsorb for 5 min. Then, 100 µL of the suspension was added to 20 mL of LB medium and cultivated in an incubator shaker. The number of infection centers was determined at times: 5, 10, 15 min after infection by mixing l0 µL of the sample with 200 µL of an overnight culture of appropriate indicator bacteria and 4 mL of top agar. To determine the number of intracellular progeny phages, samples were shaken vigorously for 1 min with equal volume of chloroform and cleared by centrifugation. The number of PFU (number of phages able to form plaques) per mL was estimated by titration using the double-layer agar plate method. Plates were incubated at 37 °C overnight. Burst size was calculated as a ratio of phage titer to the titer of infection centers. Each experiment was repeated three times.

### 2.7. Determination of the Host Range

The phage host range was determined using spot test method [22]. The bacterial isolates listed in Tables 2 and 3 were incubated overnight at 37 °C in LB liquid medium. Two hundred microliters of each test bacterial culture were added to 4 mL of molten 0.7% LB agar and the mixture was overlaid on 1.5% LB agar plates. The plates were left to solidify. Then 2.5 µL of ten-fold dilutions of each phage was spotted onto the surface of the double-layer agar plates with a tested host. Initial concentration of phages vB_SauM-A, vB_SauM-C and vB_SauM-D was 8 × 10^10^ PFU/mL, 2.4 × 10^11^ PFU/mL, 6.4 × 10^11^ PFU/mL, respectively. Following incubation at 37 °C for 24 h, the plates were examined for the presence of plaques. All the experiments were performed in triplicate.

### 2.8. Sensitivity of Phage Particles to Temperature, pH and Chloroform and Virion Stability

The effect of temperature and various pH levels was performed according to the procedure described previously [22]. For thermo-stability testing, equal volumes of bacteriophages were incubated at 40 °C (40 min) and 62 °C (40 min). After incubation, the phage titer was determined using the spot test method as described above. For pH stability testing, phage lysate was mixed (at the volume proportion 1:9) with LB medium of different pH values (2–12, adjusted using NaOH or HCl). Phage titer was assessed after 2 h incubation at 37 °C. Virion stability was assessed by titration of phage lysates over time. Purified phage lysate was stored at 10 °C and titrated over the period of 10 months in order to analyze any changes in phage titer. All the experiments were repeated three times.

### 2.9. Spectrophotometric Assay of Phage-Treated Liquid

The effects of phages on representative strains of *S. aureus* in LB medium were observed by measuring the OD_600_ with a SmartSpec 3000 spectrophotometer (BIO-RAD, Hercules, CA, USA) following the procedure described previously [23], with some modifications. Overnight cultures of the bacterial strains were diluted 1:100 in LB medium and incubated at 37 °C with shaking at 150 rpm until early logarithmic phase (OD_600_, 0.1) was reached. Then, the phage lysate was added to achieve MOI of 0.1, 0.25, 0.5, 1. The OD_600_ of each culture was measured every 15 min for a minimum of 4 h following phage addition.

### 2.10. Lysogeny Test for Verification of Lytic Cycle

Test was performed according to D’Andrea et al. [24] with minor modification. Briefly, bacterial host strain was propagated in TSB broth and infected by bacteriophages at a MOI equal to approx. 1. Cultures were incubated at 37 °C with shaking at 160 rpm for 24 h. The infected culture was then diluted and bacteriophage stock was added at an MOI > 1000 and incubated for 1 h. Subsequently, the culture was plated on TSB and incubated overnight. Single colonies were streaked and the resistance to isolated bacteriophages was tested by spotting 10 µL of phage lysate. The cells which survived this exposure were confirmed to be phage-resistant by PCR screening with primers specific to the *Kayvirus* DNA polymerase I gene.

### 2.11. Extraction of Phage DNA

The purified phage lysate (10^9^ PFU/mL) was treated with DNase I (1 U/μL; A&A Biotechnology, Gdynia, Poland) and RNase A (5 µg/μL; A&A Biotechnology, Gdynia, Poland). The mixture was incubated for 30 min at 37 °C in order to digest any exogenous DNA and RNA. Then, DNase I and RNase A were inactivated according to manufacturer protocol. The MasterPure™ Complete DNA and RNA Purification Kit (Epicentre, Madison, WI, USA) was used to isolate the genomic DNA of phages. The DNA concentration and purity was determined spectrophotometrically at 260 nm [25].

### 2.12. Phage Genome Sequencing and Bioinformatics Analysis

DNA samples were sequenced in MiSeq (Illumina, Inc., San Diego, CA, USA) genome sequencer and assembled with CLCGenomicWorkbench by the Genomed company. Open reading frames (ORFs) prediction and genome annotations were prepared with the use of Geneious Prime 2019.2.1 (https://www.geneious.com). Genome annotations were checked by sequence comparison of protein sequence using the blastn software (https://blast.ncbi.nlm.nih.gov). Whole genome-based phylogeny was prepared with the use of VICTOR software [26]. The pairwise comparisons of the bacteriophage genomes were prepared with applying the Genome-BLAST Distance Phylogeny (GBDP) method with settings optimized for bacteriophages [26,27]. Phylogeny between isolated phages was prepared with the use of CSI phylogeny 1.4 [28]. Prediction of bacteriophage life cycle was performed using the PHACTS software [29]. Sequence similarity of isolated bacteriophages was prepared with the use of Circoletto software [30]. The bacteriophages G1, K, JD007, MCE-2014 and phiIPLA-RODI (NCBI accession numbers: AY954969.1, KF766114.1, JX878671.1, KJ888149.1, KP027446.1 respectively) were used for the generation of sequence similarity graphs.

### 2.13. Statistical Analysis

The obtained data were analyzed by two-way ANOVA with Tuckey post-hoc tests. *p* values lower than 0.05 were considered statistically significant. All statistical analyses were carried out using GraphPad Prism 7 (GraphPad Software, San Diego, CA, USA). All data are presented as mean from minimum five replicates with standard deviation (SD).

## 3. Results

### 3.1. Virion Morphology

**Figure 1:**
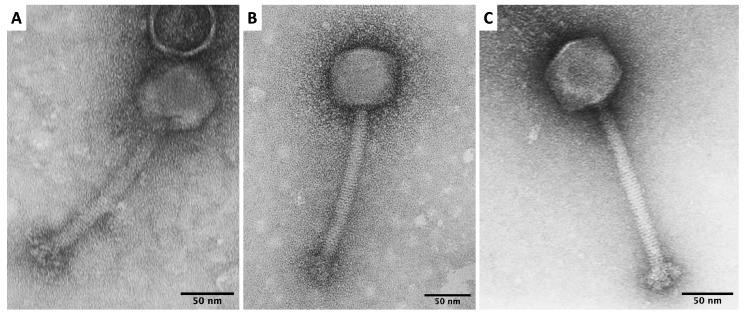
Transmission electron micrographs (TEM) of vB_SauM-A (**A**), vB_SauM-C (**B**), vB_SauM-D (**C**).

Bacteriophages were isolated from different wastewater treatment plants with initially 9 phages isolated (10.47% positive samples), 6 phages were excluded from this study according to their low lytic activity or genome characteristic showed lysogenic life cycle. The isolated phages were characterized with regard to their morphology. TEM images (Figure 1) revealed that virions of vB_SauM-A, vB_SauM-C and vB_SauM-D have icosahedral heads and long contractile tails of 149–158 nm in the extended state. Phage dimensions are summarized in Table 1. Also, the knob-like appendages, previously described by O’Flaherty et al. [31], extending from the baseplate were evident. Based on their morphology, we assumed that phages vB_SauM-A, vB_SauM-C and vB_SauM-D have myovirids morphotypes.

### 3.2. Analysis of the Bacteriophage Adsorption Rate and One-Step Growth Curves

**Figure 2:**
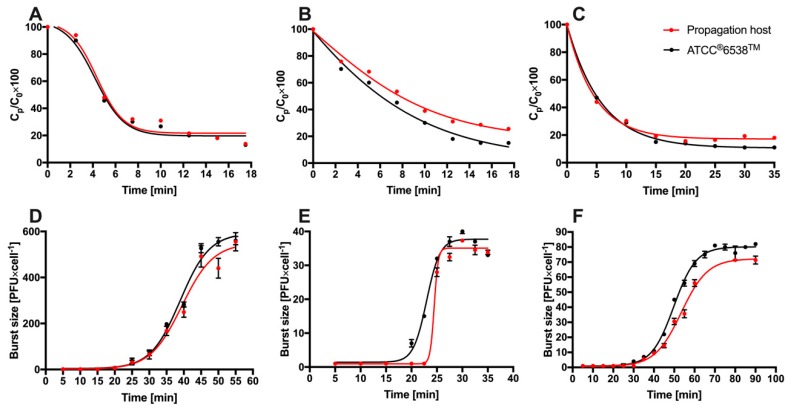
Characterizations of phages vB_SauM-A, vB_SauM-C, vB_SauM-D. Kinetics of vB_SauM-A (**A**), vB_SauM-C (**B**) and vB_SauM-D (**C**) phages adsorption on propagation and reference host cells. Curve for one-step growth of phages vB_SauM-A (**D**), vB_SauM-C (**E**) and vB_SauM-D (**F**) on propagation and reference host cells. Results are presented as mean values ± SD from three independent experiments.

The life cycle and adsorption rate of phages vB_SauM-A, vB_SauM-C and vB_SauM-D were determined on *S. aureus* 203, 343 and 342 hosts, respectively and on reference *S. aureus* ATCC^®^6538™. The adsorption curves of these bacteriophages on reference strain and propagation hosts were very similar (Figure 2) and showed that after 10 min, depending on the strain, 69–73.3%, 61–64.6%, 70–70.9% of bacteriophages vB_SauM-A, vB_SauM-C, vB_SauM-D were adsorbed. Statistical analysis revealed statistically significant difference between adsorption of vB_SauM-C and vB_SauM-A, vB_SauM-D (*p* > 0.05). There was no significant difference between vB_SauM-A and vB_SauM-D. Bacteriophage vB_SauM-A showed a short latent period of only 25 min and an extremely large burst size of approximately 500 PFU/infected cell. Differences between burst size of these phages were significant (*p* > 0.05) when compared.

### 3.3. Determination of the Host Range

The host ranges of phages vB_SauM-A, vB_SauM-C, vB_SauM-D were determined using 21 different bacterial strains that are listed in Table 2 and 67 MDRSA clinical isolates listed in Table 3. We found out that tested bacteriophages are able to efficiently infect different *S. aureus* strains and one of reference *S. epidermidis* strains but no other bacterial genera. Phages vB_SauM-A and vB_SauM-D were found to infect the same MDRSA clinical isolates.

### 3.4. Sensitivity of Phage Particles to Temperature, pH and Chloroform and Virion Stability Analysis

Phages vB_SauM-A, vB_SauM-C, vB_SauM-D were tested for their sensitivity to physical and chemical factors and the results are listed in Table 4. All of the phages showed strong resistance to CHCl_3_ and they were stable in pH range between 4 and 8. Unfortunately, exposition to pH lower than 3 and pH equal or higher than 10 resulted in loss of phage activity. Exposition of bacteriophages to higher temperature (40 °C) resulted in approx. 50% reduction of relative phage titer. Furthermore, exposition of bacteriophages to 62 °C resulted in an inactivation of all tested bacteriophages.

Virion stability tests showed no change in titer of phages vB_SauM-C, vB_SauM-D over 10-month period. In the case of phage vB_SauM-A, a drop from original titer of 1.2 × 10^11^ PFU/mL to 1.56 × 10^10^ PFU/mL was observed. However, a decrease in titer did not affect the phage performance. Therefore, all three phages are able to withstand prolonged storage periods without the loss in ability to infect their host.

### 3.5. Lysis Profiles

**Figure 3:**
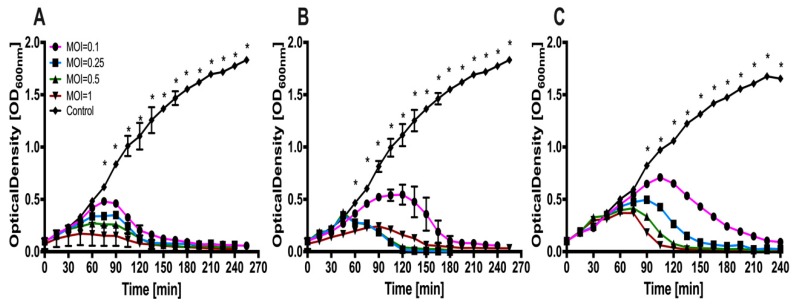
Bacteriolytic activity of phages vB_SauM-A, vB_SauM-C, vB_SauM-D at different multiplicities of infection (MOIs) (that are 0, 0.1, 0.25, 0.5 and 1) against representative MDR *S. aureus* strains in liquid culture. Each curve represents the average results for three replicates, error bars represent standard deviation (SD) (**A**) Growth curves of *S. aureus* 203 infected with vB_SauM-A (**B**) Growth curves of *S. aureus* 343 infected with vB_SauM-C (**C**) Growth curves of *S. aureus* 343 infected with vB_SauM-D. Statistically significant difference was highlighted by asterisk (*). Only those time points at which all measured optical density (OD) values were significantly different when compared to control, were indicated.

To examine the efficiency of phages vB_SauM-A, vB_SauM-C, vB_SauM-D infection, liquid culture of corresponding propagation host was infected with different MOI values and the bacterial growth was assessed by measuring the optical density of the culture. All of the isolated bacteriophages exhibit strong lytic activity, addition of phage lysate resulted in high reduction of the bacterial culture optical density. Figure 3 illustrates results of bacteriophage mediated reduction of *S. aureus* liquid culture optical density.

The lysogenization test showed limited production of bacteriophage insensitive mutants. Lack of amplicons after PCR reaction with primers specific to the *Kayvirus* DNA polymerase I gene indicate that bacteriophage do not integrate into bacterial chromosome. Bacteriophages genome analysis showed also lack of integrase genes which makes genome integration impossible.

### 3.6. Phage Genome Analysis

The complete nucleotide sequences of phages vB_SauM-A, vB_SauM-C, vB_SauM-D were determined and deposited in GeneBank under the accession numbers MN539738, MN539737, MN539736, respectively. The detailed bacteriophage genome properties are presented in Table 5. Phages have genome length between 139,031 bp and 140,086 bp with low G+C content: approximately 30%.

**Figure 4:**
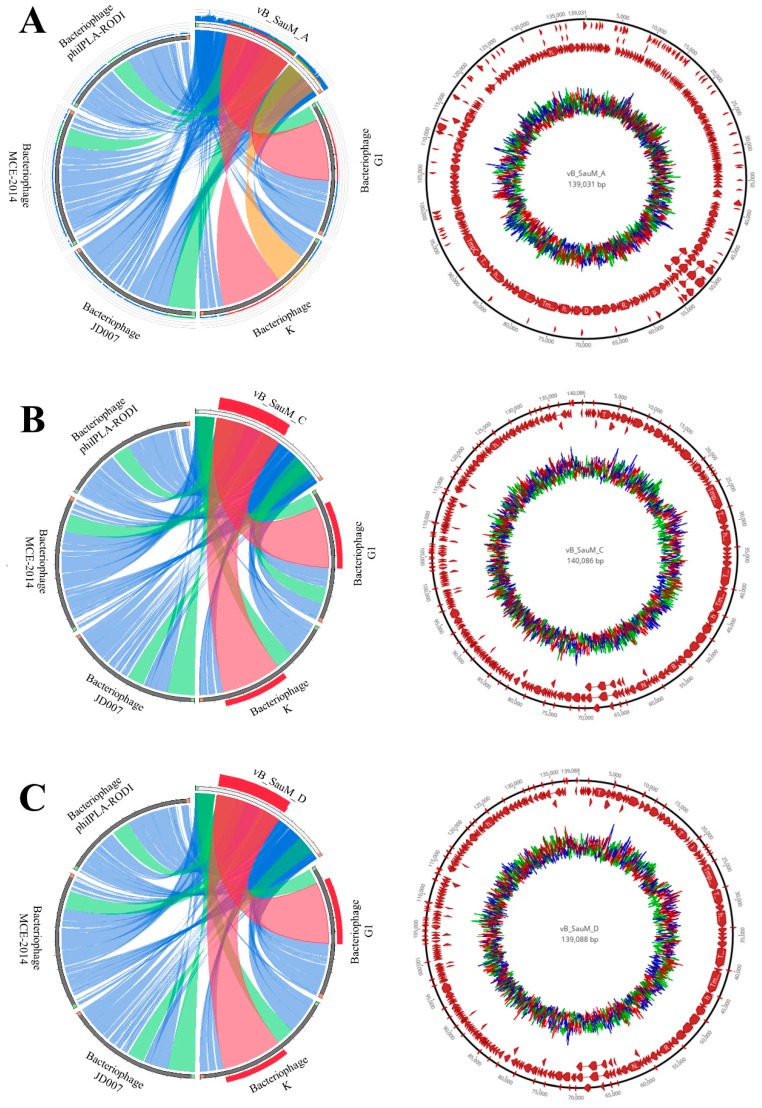
Genome organization of isolated bacteriophages. Comparison of genomes of isolated bacteriophages with selected kayviruses, where: blue represents more than 50% similarity, green more than 75% similarity, orange more than 90% similarity and the red represents more than 99% similarity. (**A**) genome similarity of bacteriophage vB_SauM-A with other kayviruses and genome organization and G+C screw of vB_SauM-A genome. (**B**) Genome similarity of bacteriophage vB_SauM-C with other kayviruses and genome organization and GC screw of vB_SauM-C genome. (**C**) genome similarity of bacteriophage vB_SauM-D with other kayviruses and genome organization and GC screw of vB_SauM-D genome.

Bacteriophages genome organization is presented in Figure 4 and supplementary Figures S1–S3. Detailed genome annotation is available in supplementary Tables S1–S3. Detailed key information that corresponds to cell lysis as well as to other aspects of phage life cycle is presented in Table 6. The table represents putative lytic genes and their products, as well as other genetic peculiarities for example, those that determine the host specificity. The middle genome regions identified in all isolated bacteriophages carry genes responsible for DNA metabolism, replication and repair. These genes are presented in DNA metabolisms and DNA replication rows of Table 6. The late genome regions are responsible for encoding the structural, cell lysis and DNA packaging proteins. The identified genes are presented in head, neck, tail, baseplate, tail fiber and lysis rows of Table 6. Additionally, four genes that encode Met, Asp, Phe and Trp tRNA were annotated. Integrases were not found in the bacteriophage genomes. Analysis showed similar genome organization of the bacteriophages vB_SauM-C and vB_SauM-D. The genome similarity of isolated bacteriophages showed 95–97% coverage with phage K-type strain of the *kayvirus*. Figure 4 also shows high similarity of isolated bacteriophages to selected kayviruses like bacteriophage G1, K, JD007, MCE-2014 and phiIPLA-RODI (NCBI accession numbers: AY954969.1, KF766114.1, JX878671.1, KJ888149.1, KP027446.1, respectively). Additionally, the prediction of bacteriophage lifecycle with PHACT software indicates that all of isolated bacteriophages are characterized by lytic life cycle.

**Figure 5:**
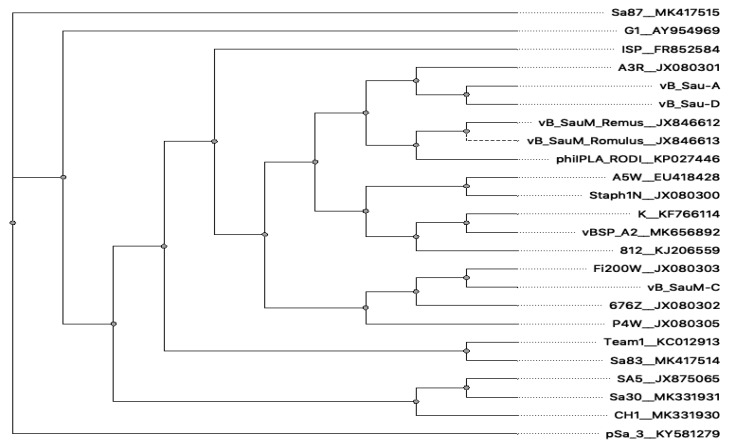
Whole genome phylogeny of selected kayviruses and newly isolated bacteriophages described in this work.

**Figure 6:**
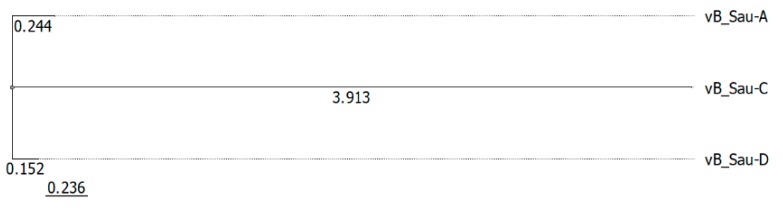
Phylogeny of newly isolated bacteriophages described in this work.

The phylogenetic analysis based on the GBDP of whole phage genomes revealed that isolated bacteriophages are related to members of the genus *Kayvirus* in subfamily *Twortvirinae* of the family *Herelleviridae* (Figure 5). Worth mentioning is that bacteriophages can be grouped in one clade with other bacteriophages isolated in Poland. Phylogenetic relative differences between the three newly isolated phages were shown in the Figure 6.

## 4. Discussion

Phage therapy is seen as a more cost-effective alternative to the isolation and development of new antibiotics against multidrug-resistant nosocomial pathogens such as MRSA [32]. Phages can be found in all habitats and be isolated relatively easy and at low cost. Furthermore the use of phages lacks serious side effects on eukaryotic cells, that some antibiotics may cause [4,32]. However, an in-depth characterization of isolated phages is required in order to assess their therapeutic potential against clinical isolates of pathogenic bacteria. In the last decade, several papers describing the isolation, biological and genomic characterization of *S. aureus* bacteriophages have been published [9,11,33]. As bacteriophages tend to be more specific than antibiotics [4] and since properties of pathogenic strains of MRSA can differ based on a variety of factors [34,35,36], there is a tendency to focus on strains and phages obtained locally [8,10,37]. So far, there is a lack of information on the bacteriophages active against clinical isolates from Gdansk region, Poland. Thus, this study aimed at characterizing three newly isolated phages that show lytic activity against clinical isolates of multidrug-resistant *Staphylococcus aureus* in a view of their potential application in therapy. Bacteriophages were characterized according to protocols implemented by other research groups [8,10,11,21] taking the phage morphology, genetics, survivability, stability, host range and growth parameters into consideration.

TEM observation revealed that all three phages have similar morphology with the same head shape, similar tail lengths and diameters. According to previously published criteria [38] phages vB_SauM-A, vB_SauM-C and vB_SauM-D display morphology corresponding to phages of the *myoviridae* family. However, taking into account genome length, low G+C content, gene organization, it can be stated that isolated viruses are similar to well-studied *S. aureus* phages such as phage K, G1 or ISP, which have been applied successfully in the treatment of bacterial infections [31,39]. Comparative genomic analysis of isolated bacteriophages showed that these bacteriophages are closely related to bacteriophage K and other kayviruses. Phylogenetically isolated bacteriophages are closely related to other bacteriophages isolated in Poland and widely described by Łobocka et al. [40]. Considering the latest changes in phage taxonomy [41] we conclude that phages vB_SauM-A, vB_SauM-C and vB_SauM-D belong to *Kayvirus* genus of *Herelleviridae* family. Additionally, comparison of genomes structure of isolated bacteriophages to well-known viruses representing *Kayvirus* genus showed high similarity of genome structure of isolated bacteriophages to kayviruses. It is worth mentioning that bacteriophage lifestyle prediction showed strictly virulent cycle also characteristic to other kayviruses and there were no integrases detected in these bacteriophages’ genomes. Lysins gene (*lysK*) of isolated bacteriophages showed high similarity to *lysK* gene and lysins of bacteriophage LM12, according to which there is high probability that bacteriophages will exhibit biofilm reducing properties [9,42].

Bacteriophages can be resistant to various unfavorable environmental conditions [18,43]. Based on phage tolerance to various environmental factors as well as other properties their therapeutic potential can be assessed [22]. Performed experiments revealed that isolated phages were resistant to pH between 4 and 8, which is a typical range of pH tolerance for most of environmental phages [22,44,45]. Unfortunately, all of isolated bacteriophages showed limited resistance to the growth temperature. None of them is able to persist 40 min at 62 °C. These results seem to be similar to the results of Zhang et al. (2015) which showed that other *Kayvirus,* bacteriophage JS25, rapidly decreased its viability when it to 60 °C [43]. However, since isolated phages are considered to be used as a potential treatment against MDRSA, primary focus should be on their performance and survivability at 37 °C, which has been proven to be satisfactory. Isolated phages are also able to survive long storage periods without decrease in titer or activity, which is also a desirable feature of phages used for therapy [45,46,47].

The spot test results indicate that phages vB_SauM-A, vB_SauM-C, vB_SauM-D are specific to *Staphylococcus* sp. and do not possess the ability to infect other bacterial genera both Gram-negative (such as *Salmonella*, *Shigella* or *Pseudomonas*) and Gram-positive (*Lactobacillus*, *Enterococcus*). Furthermore, it was found that isolated phages have a wide host range among MDRSA isolates collected from different clinical sites, both from inpatients and outpatients which is in accordance with other staphylococcal K-like phages [9,16,18]. Though it was proven that selected spot test method, when spotting high titer lysates (10^9^ PFU/mL), can generate false positive results correlated with abortive infection, lysis from without, and enzyme activity [48] it is still used to screen the bactericidal ability of the isolated phages [25,49,50]. Moreover, applying phages at lower concentrations prevents from the overestimation of phage sensitivity [51]. In this study we used 10-fold dilutions of phage lysates (from 10^11^ PFU/mL to 10^2^ PFU/mL) to ensure that a productive phage infection occurred. Broad lytic spectrum on pathogenic strains of *S. aureus* and high specificity of isolated phages show that they can be considered a promising tool for therapeutic purposes [9].

The one-step growth experiments displayed that burst size of vB_SauM-C and vB_SauM-D can be compared to other phages but the burst size of vB_SauM-A is considerably higher than that obtained for other anti-staphylococcal bacteriophages like JS25, SAP-26, phage K and DRA88 which produced approx. 21, 107, 125 or 76 PFU per infected cell, respectively [18,43,52]. Broad host range in combination with short latent period and a relatively large burst size confirm the lytic nature of the isolated bacteriophage.

The lytic potential of isolated viruses showed that all isolated bacteriophages possess strong lytic activity. The bacteriolytic assay showed that all of isolated bacteriophages were able to completely lyse *S. aureus* in in vitro culture at a MOI approx. 0.1. In contrast, bacteriophage JS25 completely lyse the bacterial culture at a MOI = 1 but the other MOI values were not shown [53]. The specific and efficient lysis of the host cell showed that these bacteriophages have a feature that make them potentially good candidates for therapeutic purposes. Additionally, low phage insensitive mutant production, bacteriophage impossibility to integrate into the host genome, lack of *Staphylococcus* specific DNA and toxins in genome, minimizes the transduction probability.

Taken together, the isolated bacteriophages demonstrate properties desirable for therapeutic phages. Phage vB_SauM-A with its high lytic potential and unusually large burst size shows the most promise in that aspect and its potential should be further investigated in future research.

## Figures and Tables

**Table 1:** Dimensions of phages vB_SauM-A, vB_SauM-C and vB_SauM-D obtained from transmission electron micrographs.

Parameter	vB_SauM-A	vB_SauM-C	vB_SauM-D
Head morphology	Icosahedral	Icosahedral	Icosahedral
Head length (nm)	62.99 ± 9.99	76.75 ± 5.04	72.80 ± 3.66
Head diameter (nm)	63.33 ± 5.81	68.37 ± 4.14	65.98 ± 3.11
Tail length (nm)	149.70 ± 14.65	153.51 ± 15.16	158.05 ± 6.56

**Table 2:** Host range of phages vB_SauM-A, vB_SauM-C, vB_SauM-D on different bacterial species.

Species	Reference Number	vB_SauM-A	vB_SauM-C	vB_SauM-D
*Staphylococcus epidermidis*	ATCC 12228	−	−	−
*Staphylococcus epidermidis*	ATCC 14990	++	+	++
*Staphylococcus intermedius*	PCM 2405 *	−	−	−
*Staphylococcus aureus*	ATCC 25923	++	+++	++
*Staphylococcus aureus*	ATCC 29213	++	++	++
*Escherichia coli*	ATCC 11775	−	−	−
*Salmonella Typhimurium*	ATCC 14028	−	−	−
*Salmonella Enteritidis*	ATCC 13076	−	−	−
*Shigella flexneri*	ATCC 12022	−	−	−
*Shigella sonnei*	ATCC 25931	−	−	−
*Proteus vulgaris*	ATCC 6380	−	−	−
*Proteus mirabilis*	ATCC 7002	−	−	−
*Yersinia enterocolitica*	ATCC 27729	−	−	−
*Pseudomonas aeruginosa*	ATCC 27853	−	−	−
*Enterococcus faecalis*	ATCC 29212	−	−	−
*Lactococcus lactis*	ATCC 19435	−	−	−
*Lactobacillus gasseri*	ATCC 19992	−	−	−
*Lactobacillus acidophilus*	ATCC 4356	−	−	−
*Bacillus cereus*	ATCC 14579	−	−	−
*Streptococcus agalactiae*	ATCC 12386	−	−	−
*Listeria monocytogenes*	ATCC 19118	−	−	−

* PCM, Polish Collection of Microorganisms. +++ plaques at lysate concentration ≤ 10^4^ PFU/mL, ++ plaques at lysate concentration 10^5^–10^6^ PFU/mL, + plaques at lysate concentration 10^7^ PFU/mL, − no plaques or plaques at lysate concentration ≥10^8^ PFU/mL.

**Table 3:** Host range of phages vB_SauM-A, vB_SauM-C, vB_SauM-D on 67 multidrug resistant *S. aureus* clinical isolates.

SA ^1^	Origin ^2^		Antibiotic Resistance ^3^										A ^4^	C ^5^	D ^6^
4	IP	Pus	Fox	P	E	CC			SXT	TE			−	−	−
17	IP	Wound	Fox	P	E	CC			SXT	TE			−	−	−
19	IP	Blood	Fox	P	E	CC	NOR	CIP	SXT	TE			−	−	−
20	IP	Wound	Fox	P	E	CC			SXT	TE			−	−	−
21	IP	drain content	Fox	P		CC				TE			−	−	−
23	IP	Wound	Fox	P	E	CC	NOR	CIP	SXT	TE			−	−	−
44	IP	Throat	Fox	P	E	CC	NOR	CIP		TE			+++	−	+++
70	IP	Throat	Fox	P	E	CC	NOR	CIP		TE			++	+++	+++
108	IP	Urine	Fox	P	E	CC	NOR	CIP					+++	+	+++
109	IP	Wound	Fox	P	E	CC	NOR	CIP					++	+	+++
110	IP	Fistula	Fox	P	E	CC	NOR	CIP					++	+	+++
111	IP	Wound	Fox	P	E	CC	NOR	CIP					++	+++	+++
112	IP	Bedsore	Fox	P	E	CC	NOR	CIP		TE		GM	−	−	−
113	IP	Wound	Fox	P	E	CC	NOR	CIP			C		++	+++	+++
115	IP	Wound	Fox	P	E	CC	NOR	CIP					++	+	+++
116	IP	wound	Fox	P	E	CC	NOR	CIP					++	+	+++
118	IP	blood	Fox	P	E	CC	NOR	CIP		TE		GM	−	+	−
120	IP	bedsore	Fox	P	E	CC	NOR	CIP		TE		GM	−	+	−
121	IP	bronchial tree discharge	Fox	P	E	CC	NOR	CIP		TE		GM	−	+	−
122	IP	bronchial tree discharge	Fox	P	E	CC	NOR	CIP					+++	+++	+++
124	IP	bronchial tree discharge	Fox	P	E	CC	NOR	CIP					+++	+++	+++
126	OP	sputum	Fox	P	E	CC	NOR	CIP		TE		GM	−	+	−
140	OP	furuncle	Fox	P	E	CC	NOR	CIP		TE	C		−	−	−
149	IP	nose	Fox	P	E	CC	NOR	CIP					++	+	+++
151	IP	furuncle	Fox	P	E	CC					C		−	+	−
173	OP	furuncle	Fox	P	E	CC				TE	C		+	+	−
182	OP	furuncle	Fox	P	E	CC					C		−	+	−
183	OP	furuncle	Fox	P	E	CC				TE	C		−	+	−
184	IP	wound	Fox	P	E	CC	NOR	CIP		TE		GM	−	+	+
193	IP	bedsore	Fox	P	E	CC	NOR	CIP					++	+++	+++
196	OP	wound	Fox	P	E	CC				TE	C		−	−	−
198	OP	wound	Fox	P	E	CC	NOR	CIP		TE	C	GM	−	+	+
199	OP	wound	Fox	P	E	CC				TE	C		+++	+++	+++
200	OP	sputum	Fox	P	E	CC	NOR	CIP					+++	+	+++
201	IP	sputum	Fox	P	E	CC	NOR	CIP					−	+	+
202	IP	bronchial tree discharge	Fox	P	E	CC	NOR	CIP					+++	+++	+++
203	IP	vascular catheter	Fox	P	E	CC	NOR	CIP					+++	+++	+++
204	OP	wound	Fox	P	E	CC							−	−	−
205	OP	bedsore	Fox	P	E	CC	NOR	CIP					−	+	+
258	IP	nose	Fox	P	E	CC	NOR	CIP					+++	+++	+++
271	IP	wound	Fox	P	E	CC	NOR	CIP			C		+++	+++	+++
297	IP	sore	Fox	P	E	CC	NOR	CIP			C		+++	+++	+++
298	IP	tissue	Fox	P	E	CC	NOR	CIP					+++	+++	+++
301	IP	ear	Fox	P	E	CC	NOR	CIP					+++	++	+++
305	IP	wound	Fox	P	E	CC	NOR	CIP					+++	+++	+++
311	IP	wound	Fox	P	E	CC	NOR	CIP					+++	++	+++
315	IP	pus from abscess	Fox	P	E	CC				TE	C		−	+	−
316	IP	wound	Fox	P	E		NOR	CIP					+++	+	+++
317	IP	skin	Fox	P	E		NOR	CIP					+++	+	+++
324	IP	nose	Fox	P	E	CC	NOR	CIP					+++	++	+++
340	IP	wound	Fox	P	E	CC	NOR	CIP					+++	++	+++
341	IP	urine	Fox	P	E		NOR	CIP					++	+	+++
342	IP	endotracheal tube content	Fox	P	E	CC	NOR	CIP					+++	+++	+++
343	IP	wound	Fox	P	E	CC	NOR	CIP					+++	+++	+++
344	IP	wound	Fox	P	E	CC					C		−	+	−
345	IP	nose	Fox	P	E	CC					C		−	+	−
351	IP	pus from abscess	Fox	P	E	CC					C		−	+	−
352	IP	nose	Fox	P	E	CC	NOR	CIP					+++	+++	+++
353	IP	nose	Fox	P	E	CC	NOR	CIP					+++	+++	+++
355	IP	endotracheal tube content	Fox	P	E	CC	NOR	CIP					+++	+++	+++
357	IP	ear	Fox	P			NOR	CIP					+++	+++	+++
358	IP	bronchial aspirate	Fox	P	E	CC	NOR	CIP			C		+++	+++	+++
366	IP	wound	Fox	P	E	CC	NOR	CIP					++	+++	+++
367	IP	wound	Fox	P	E	CC	NOR	CIP					+++	+++	+++
369	IP	nose	Fox	P	E		NOR	CIP					−	+	−
370	IP	endotracheal tube content	Fox	P	E	CC	NOR	CIP					++	+++	+++
371	IP	bronchoalveolar lavage fluid	Fox	P						TE	C		+++	+++	++

^1^ SA, *Staphylococcus aureus* strain; ^2^ IP, inpatient, OP, outpatient; ^3^ FOX, cefoxitin (30 μg); P, penicillin (10 U); E, erythromycin (15 μg); CC, clindamycin (2 μg); NOR, norfloxacin (10 μg); CIP, ciprofloxacin (5 μg); SXT, sulfamethoxazole/trimethoprim (23.75 μg/1.25 μg); TE, tetracycline (30 μg); C, chloramphenicol (30 μg); GM, gentamicin (10 μg); ^4^ vB_SauM-A; ^5^ vB_SauM-C; ^6^ vB_SauM-D; +++ plaques at lysate concentration ≤ 10^4^ PFU/mL, ++ plaques at lysate concentration 10^5^–10^6^ PFU/mL, + plaques at lysate concentration 10^7^ PFU/mL, − no plaques or plaques at lysate concentration ≥10^8^ PFU/mL.

**Table 4:** Bacteriophages sensitivity to physical and chemical factors.

Phage Name	Phage Survivability in Studied Conditions (Relative Phage Titer in %)									
	40 °C (40 min)	62 °C (40 min)	pH 2 (2 h)	pH 3 (2 h)	pH 4 (2 h)	pH 6 (2 h)	pH 8 (2 h)	pH 10 (2 h)	pH 12 (2 h)	CHCl_3_ (4 °C; 1 h)
**vB_SauM-A**	58	0	0	0	8	100	83	0	0	97
**vB_SauM-C**	59	0	0	0	20	100	100	0	0	80
**vB_SauM-D**	43	0	0	0	87	100	95	0	0	100

**Table 5:** Bacteriophages genomes properties.

Phage Name	Genome Properties		
	Genome Length	GC content (%)	Phage K Similarity (%)
**vB_SauM-A**	139,031	30.45	97
**vB_SauM-C**	140,086	30.43	95
**vB_SauM-D**	139,088	30.46	97

**Table 6:** Detailed information on bacteriophages.

Gene Group	vB_SauM-A	vB_SauM-C	vB_SauM-D
*DNA metabolisms*	nucleotide kinase (orf613), NTP pyrophosphohydrolase (orf612), nucleoside 2-deoxyribosyltransferase (orf602), RNA ligase (orf601), ribonuclease H (orf559), HNH endonuclease (orf587, 580), intron-encoded nuclease (orf1593), endonuclease (orf201, 264), homing endonuclease (orf206), ribose-phosphate pyrophosphokinase (orf280)	HNH endonuclease (orf312,319, 392, 688), ribonuclease H (orf2411), NTP pyrophosphohydrolase (orf343) nucleotide kinase (orf344), ribose-phosphate pyrophosphokinase (orf187), intron-encoded nuclease (orf216),	ribonuclease H (orf594), HNH endonuclease (orf580, orf587), RNA ligase (orf601), NTP pyrophosphohydrolase (orf612), ribonuclease H (orf453), intron-encoded nuclease (orf592),
*DNA replication*	DNA helicase (orf177, 180), recombination-related endonuclease (orf181, 184), anti-sigma factor (orf186), DNA primase (orf187), resolvase (orf190), ribonucleotide reductase stimulatory protein (orf191), ribonucleotide reductase large subunit (orf193), ribonucleotide reductase small subunit (orf195), DNA polymerase I (orf208), recombinase (orf218), sigma factor (orf221), DNA repair exonuclease (orf230), sliding clamp inhibitor (orf252), nucleotidyl transferase (orf642), terminase large subunit (orf101)	nucleotidyl transferase (orf373), recombinase (orf125), endonuclease (orf669), DNA polymerase I (orf107), ribonucleotide reductase large subunit (orf100), ribonucleotide reductase small subunit (orf638), resolvase (orf97), DNA primase (orf94), anti-sigma factor (orf93), recombination-related endonuclease (orf91, 88), helicase (orf87, 84), terminase large subunit (orf8)	DNA polymerase I (orf200), anti-sigma factor (orf186), recombination-related endonuclease (orf181), sigma factor (orf221), DNA helicase (orf177, 180), DNA primase (orf187), DNA repair exonuclease (orf230), ribonucleotide reductase small subunit (orf227), terminase large subunit (orf101), ribonucleotide reductase large subunit (orf193), recombinase (orf218)
*Head*	portal protein (orf110), prohead protease (orf112), major capsid protein (orf115)	major capsid protein (orf22), prohead protease (orf19), portal protein (orf17), putative virion protein (orf49)	portal protein (orf110), major capsid protein (orf115), prohead protease (orf112)
*neck protein*	neck protein (orf120, 122)	putative neck protein (orf29, 27)	neck protein (orf120, 122)
*Tail*	major tail protein (orf38), tail sheath protein (orf127), tail tube protein (orf129), tail assembly chaperone(orf137), tape-measure protein (orf140), tail murein hydrolase (orf145), cysteine protease (orf147), tail protein (orf226)	major tail protein (orf366), tail protein (orf 133), tail central spike (orf55), tail murein hydrolase (orf52), tape measure protein (orf47), tail morphogenetic protein (orf241), tail assembly chaperone (orf44), tail tube protein (orf36), tail sheath protein (orf34), baseplate hub assembly protein (orf31)	major tail protein (orf38), tape-measure protein (orf140), tail murein hydrolase (orf145), nicotinamide phosphoribosyl transferase (orf381), tail morphogenetic protein (orf55), adsorption-associated tail protein (orf162), tail assembly chaperone (orf137), tail sheath (orf127), tail tube protein (orf129), receptor binding protein (orf174, 170), tail central spike (orf148)
*baseplate*	baseplate component (orf151, 155, 173), baseplate wedge subunit (orf154), baseplate protein (orf160), receptor binding protein (orf170), oxidoreductase (orf197)	putative baseplate component (orf80, 60, 58), receptor binding protein (orf81), receptor binding protein (orf77) baseplate protein (orf67) baseplate component (orf62), baseplate wedge subunit (orf61)	peptidoglycan binding protein (orf604), baseplate component (orf153, 155,160, 173), baseplate wedge subunit (orf154), baseplate hub assembly protein (orf124)
*Tail fiber*	tail fiber protein (orf169)	tail fiber protein (orf69), Tail fiber protein complex (orf65)	tail fiber protein (orf169)
*Lysis*	lytic transglycosylase (orf588), *N*-acetylmuramoyl-*L*-alanine amidase (orf578), holin (orf575)	holin (orf307), *N*-acetylmuramoyl-*L*-alanine amidase (orf313), lytic transglycosylase (orf320)	holin (orf575), *N*-acetylmuramoyl-*L*-alanine amidase (orf581, 578) lytic transglycosylase (orf588)

## Supplementary Materials

The following are available online at https://www.mdpi.com/2076-2607/7/10/471/s1, Figure S1: Genome organization of bacteriophage vB_SauM_A, Table S1: Genome annotation of bacteriophage vB_SauM_A, Figure S2: Genome organization of bacteriophage vB_SauM_C, Table S2: Genome annotation of bacteriophage vB_SauM_C, Figure S3: Genome organization of bacteriophage vB_SauM_D, Table S3: Genome annotation of bacteriophage vB_SauM_D.
