# Supplementary material for: Characterization of the Three New Kayviruses and Their Lytic Activity Against Multidrug-Resistant Staphylococcus aureus
Source: Microorganisms. 2019 Oct 18;7(10):471. doi: 10.3390/microorganisms7100471 (PMC6843549; doi:10.3390/microorganisms7100471)
Supplement: Supplementary file 1 [file microorganisms-07-00471-s001.pdf]

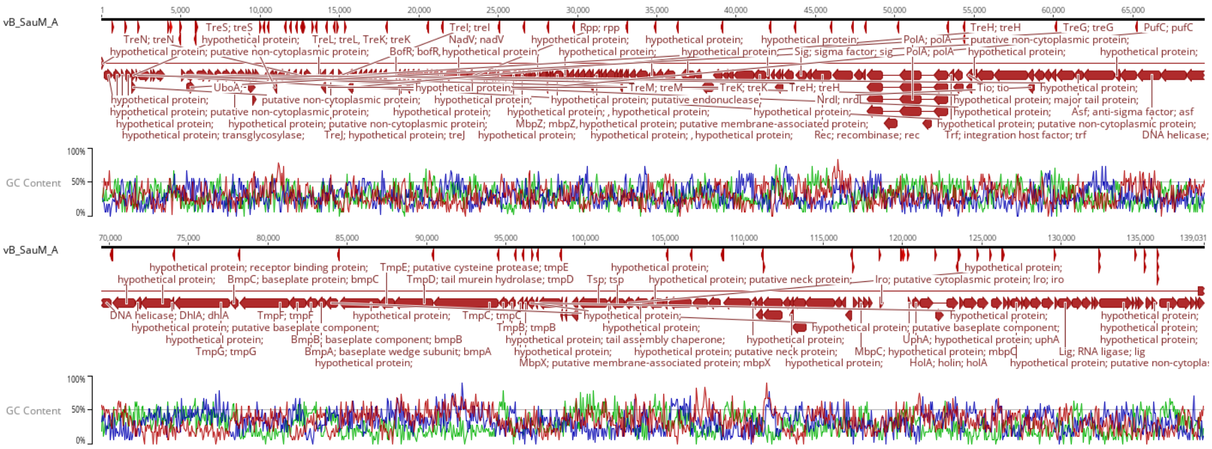

Figure S1. Genome organization of bacteriophage vB_SauM_A

Table S1. Genome annotation of bacteriophage vB_SauM_A

Name	gene	product	Type	Minimum	Maximum	Length	# Intervals	Direction
**anti-sigma factor CDS; asf CDS**	asf	Asf; anti-sigma factor	CDS	61315	61911	597	1	Reverse
**baseplate wedge subunit CDS; bmpA CDS**	bmpA	BmpA; baseplate wedge subunit	CDS	82385	83089	705	1	Reverse
**baseplate component CDS; bmpB CDS**	bmpB	BmpB; baseplate component	CDS	81324	82370	1047	1	Reverse
**baseplate protein CDS; bmpC CDS**	bmpC	BmpC; baseplate protein	CDS	77612	78133	522	1	Reverse
**bofL CDS; putative non-cytoplasmic protein CDS**	bofL	BofL; putative non-cytoplasmic protein	CDS	9704	9940	237	1	Forward
**bofR CDS**	bofR	BofR	CDS	18530	18631	102	1	Reverse
**dmcA CDS; putative membrane-associated protein CDS**	dmcA	DmcA; putative membrane-associated protein	CDS	117559	117891	333	1	Forward
**dmcB CDS; virion component CDS**	dmcB	DmcB; virion component	CDS	117904	118113	210	1	Forward
**dmd CDS; putative cytoplasmic protein CDS**	dmd	Dmd; putative cytoplasmic protein	CDS	116138	116416	279	1	Reverse
**DNA helicase CDS; dhlA CDS**	dhlA	DNA helicase; DhlA	CDS	68383	70131	1749	1	Reverse
**DNA helicase CDS; dhlB CDS**	dhlB	DNA helicase; DhlB	CDS	65323	66765	1443	1	Reverse
**DNA polymerase I CDS**		DNA polymerase I	CDS	48237	53335	3219	3	Reverse
**DNA polymerase I CDS**		DNA polymerase I	CDS	48237	53335	3219	3	Reverse
**DUF1024 domain protein CDS**		DUF1024 domain protein	CDS	9273	9527	255	1	Forward
**hmzG CDS; putative NTP pyrophosphohydrolase CDS**	hmzG	HmzG; putative NTP pyrophosphohydrolase	CDS	134893	135195	303	1	Forward
**HNH endonuclease CDS; ksaI CDS**	ksaI	HNH endonuclease; KsaI	CDS	122090	122590	501	1	Forward
**HNH homing endonuclease CDS; I-KsaIII CDS; ksaIII CDS**	I-KsaIII; ksaIII	HNH homing endonuclease; HnhC; KsaIII	CDS	49331	50140	810	1	Reverse
**holA CDS; holin CDS**	holA	HolA; holin	CDS	120624	121127	504	1	Forward
**hypothetical protein CDS**		hypothetical protein	CDS	138639	139031	393	1	Forward
**hypothetical protein CDS**		hypothetical protein	CDS	137346	138191	846	1	Forward
**hypothetical protein CDS**		hypothetical protein	CDS	136216	137334	1119	1	Forward
**hypothetical protein CDS**		hypothetical protein	CDS	135737	136063	327	1	Forward
**hypothetical protein CDS**		hypothetical protein	CDS	132452	134500	2049	1	Forward
**hypothetical protein CDS**		hypothetical protein	CDS	113085	113906	822	1	Reverse
**hypothetical protein CDS**		hypothetical protein	CDS	112925	113107	183	1	reverse
**hypothetical protein CDS**		hypothetical protein	CDS	110417	110788	372	1	reverse
**hypothetical protein CDS**		hypothetical protein	CDS	106786	107736	951	1	reverse
**hypothetical protein CDS**		hypothetical protein	CDS	104891	105187	297	1	reverse
**hypothetical protein CDS**		hypothetical protein	CDS	102458	103078	621	1	reverse
**hypothetical protein CDS**		hypothetical protein	CDS	101603	102439	837	1	reverse

**hypothetical protein CDS**		hypothetical protein	CDS	101386	101601	216	1	reverse
**hypothetical protein CDS**		hypothetical protein	CDS	96845	96985	141	1	reverse
**hypothetical protein CDS**		hypothetical protein	CDS	96344	96802	459	1	reverse
**hypothetical protein CDS**		hypothetical protein	CDS	95744	96055	312	1	reverse
**hypothetical protein CDS**		hypothetical protein	CDS	84511	87057	2547	1	reverse
**hypothetical protein CDS**		hypothetical protein	CDS	83089	83613	525	1	reverse
**hypothetical protein CDS**		hypothetical protein	CDS	73926	74084	159	1	reverse
**hypothetical protein CDS**		hypothetical protein	CDS	70223	71599	1377	1	reverse
**hypothetical protein CDS**		hypothetical protein	CDS	66758	68371	1614	1	reverse
**hypothetical protein CDS**		hypothetical protein	CDS	63830	64207	378	1	reverse
**hypothetical protein CDS**		hypothetical protein	CDS	59828	60166	339	1	reverse
**hypothetical protein CDS**		hypothetical protein	CDS	59376	59828	453	1	reverse
**hypothetical protein CDS**		hypothetical protein	CDS	54833	55162	330	1	reverse
**hypothetical protein CDS**		hypothetical protein	CDS	53726	54322	597	1	reverse
**hypothetical protein CDS**		hypothetical protein	CDS	53726	54286	561	1	reverse
**hypothetical protein CDS**		hypothetical protein	CDS	47926	48168	243	1	reverse
**hypothetical protein CDS**		hypothetical protein	CDS	47427	47909	483	1	reverse
**hypothetical protein CDS**		hypothetical protein	CDS	46069	47340	1272	1	reverse
**hypothetical protein CDS**		hypothetical protein	CDS	44396	44749	354	1	reverse
**hypothetical protein CDS**		hypothetical protein	CDS	41854	42114	261	1	reverse
**hypothetical protein CDS**		hypothetical protein	CDS	41095	41850	756	1	reverse
**hypothetical protein CDS**		hypothetical protein	CDS	38572	39108	537	1	reverse
**hypothetical protein CDS**		hypothetical protein	CDS	37812	38579	768	1	reverse
**hypothetical protein CDS**		hypothetical protein	CDS	37388	37834	447	1	reverse
**hypothetical protein CDS**		hypothetical protein	CDS	36525	37388	864	1	reverse
**hypothetical protein CDS**		hypothetical protein	CDS	35422	36153	732	1	reverse
**hypothetical protein CDS**		hypothetical protein	CDS	34946	35404	459	1	reverse
**hypothetical protein CDS**		hypothetical protein	CDS	34438	34881	444	1	reverse
**hypothetical protein CDS**		hypothetical protein	CDS	33717	34421	705	1	reverse
**hypothetical protein CDS**		hypothetical protein	CDS	32868	33110	243	1	reverse
**hypothetical protein CDS**		hypothetical protein	CDS	32702	32863	162	1	reverse
**hypothetical protein CDS**		hypothetical protein	CDS	31347	31523	177	1	reverse
**hypothetical protein CDS**		hypothetical protein	CDS	31058	31354	297	1	reverse
**hypothetical protein CDS**		hypothetical protein	CDS	30447	30815	369	1	reverse
**hypothetical protein CDS**		hypothetical protein	CDS	30087	30434	348	1	reverse
**hypothetical protein CDS**		hypothetical protein	CDS	29434	29739	306	1	reverse
**hypothetical protein CDS**		hypothetical protein	CDS	29069	29419	351	1	reverse
**hypothetical protein CDS**		hypothetical protein	CDS	28274	28453	180	1	reverse
**hypothetical protein CDS**		hypothetical protein	CDS	28047	28145	99	1	reverse
**hypothetical protein CDS**		hypothetical protein	CDS	27351	27644	294	1	reverse
**hypothetical protein CDS**		hypothetical protein	CDS	26133	26396	264	1	reverse
**hypothetical protein CDS**		hypothetical protein	CDS	25813	26130	318	1	reverse
**hypothetical protein CDS**		hypothetical protein	CDS	25132	25812	681	1	reverse
**hypothetical protein CDS**		hypothetical protein	CDS	24650	24850	201	1	reverse
**hypothetical protein CDS**		hypothetical protein	CDS	23959	24267	309	1	reverse
**hypothetical protein CDS**		hypothetical protein	CDS	21243	21488	246	1	reverse

**hypothetical protein CDS**		hypothetical protein	CDS	20831	21223	393	1	reverse
**hypothetical protein CDS**		hypothetical protein	CDS	20608	20829	222	1	reverse
**hypothetical protein CDS**		hypothetical protein	CDS	20231	20542	312	1	reverse
**hypothetical protein CDS**		hypothetical protein	CDS	19719	20228	510	1	reverse
**hypothetical protein CDS**		hypothetical protein	CDS	19388	19717	330	1	reverse
**hypothetical protein CDS**		hypothetical protein	CDS	19188	19397	210	1	reverse
**hypothetical protein CDS**		hypothetical protein	CDS	19188	19382	195	1	reverse
**hypothetical protein CDS**		hypothetical protein	CDS	18850	19164	315	1	reverse
**hypothetical protein CDS**		hypothetical protein	CDS	18668	18856	189	1	reverse
**hypothetical protein CDS**		hypothetical protein	CDS	18668	18835	168	1	reverse
**hypothetical protein CDS**		hypothetical protein	CDS	9534	9713	180	1	forward
**hypothetical protein CDS**		hypothetical protein	CDS	9534	9704	171	1	forward
**hypothetical protein CDS**		hypothetical protein	CDS	7117	7605	489	1	forward
**hypothetical protein CDS**		hypothetical protein	CDS	5348	5902	555	1	forward
**hypothetical protein CDS**		hypothetical protein	CDS	2170	2274	105	1	forward
**hypothetical protein CDS**		hypothetical protein	CDS	1910	2158	249	1	forward
**hypothetical protein CDS**		hypothetical protein	CDS	1256	1429	174	1	forward
**hypothetical protein CDS**		hypothetical protein	CDS	733	1215	483	1	forward
**hypothetical protein CDS; major tail protein CDS**		hypothetical protein; major tail protein	CDS	5015	5332	318	1	forward
**hypothetical protein CDS; nucleoside 2-deoxyribosyltransferase CDS**		hypothetical protein; nucleoside 2-deoxyribosyltransferase	CDS	130717	131343	627	1	forward
**hypothetical protein CDS; nucleotidyl transferase CDS**		hypothetical protein; nucleotidyl transferase	CDS	7617	8159	543	1	forward
**hypothetical protein CDS; peptidoglycan binding protein CDS**		hypothetical protein; peptidoglycan binding protein	CDS	131921	132094	174	1	forward
**hypothetical protein CDS; putative baseplate component CDS**		hypothetical protein; putative baseplate component	CDS	83613	84404	792	1	reverse
**hypothetical protein CDS; putative baseplate component CDS**		hypothetical protein; putative baseplate component	CDS	71606	71980	375	1	reverse
**hypothetical protein CDS; putative capsid component CDS**		hypothetical protein; putative capsid component	CDS	42987	43619	633	1	reverse
**hypothetical protein CDS; putative cytoplasmic protein CDS**		hypothetical protein; putative cytoplasmic protein	CDS	126393	126620	228	1	forward
**hypothetical protein CDS; putative cytoplasmic protein CDS**		hypothetical protein; putative cytoplasmic protein	CDS	6706	7104	399	1	forward
**hypothetical protein CDS; putative cytoplasmic protein CDS**		hypothetical protein; putative cytoplasmic protein	CDS	3064	3258	195	1	forward
**hypothetical protein CDS; putative DNA repair exonuclease CDS**		hypothetical protein; putative DNA repair exonuclease	CDS	39852	41102	1251	1	reverse
**hypothetical protein CDS; putative endonuclease CDS**		hypothetical protein; putative endonuclease	CDS	28467	29069	603	1	reverse
**hypothetical protein CDS; putative HNH endonuclease CDS**		hypothetical protein; putative HNH endonuclease	CDS	124769	125404	636	1	forward
**hypothetical protein CDS; putative membrane-associated protein CDS**		hypothetical protein; putative membrane-associated protein	CDS	32699	32863	165	1	reverse
**hypothetical protein CDS; putative membrane-associated protein CDS**		hypothetical protein; putative membrane-associated protein	CDS	32533	32712	180	1	reverse
**hypothetical protein CDS; putative membrane-associated protein CDS**		hypothetical protein; putative membrane-associated protein	CDS	26667	26930	264	1	reverse
**hypothetical protein CDS; putative neck protein CDS**		hypothetical protein; putative neck protein	CDS	103970	104878	909	1	reverse
**hypothetical protein CDS; putative neck protein CDS**		hypothetical protein; putative neck protein	CDS	103078	103956	879	1	reverse
**hypothetical protein CDS; putative non-cytoplasmicprotein CDS**		hypothetical protein; putative non-cytoplasmic protein	CDS	134705	134893	189	1	forward
**hypothetical protein CDS; putative non-cytoplasmicprotein CDS**		hypothetical protein; putative non-cytoplasmic protein	CDS	134500	134661	162	1	forward
**hypothetical protein CDS; putative non-cytoplasmicprotein CDS**		hypothetical protein; putative non-cytoplasmic protein	CDS	132111	132374	264	1	forward
**hypothetical protein CDS; putative non-cytoplasmicprotein CDS**		hypothetical protein; putative non-cytoplasmic protein	CDS	127507	127698	192	1	forward
**hypothetical protein CDS; putative non-cytoplasmicprotein CDS**		hypothetical protein; putative non-cytoplasmic protein	CDS	126843	127484	642	1	forward

**hypothetical protein CDS; putative non-cytoplasmicprotein CDS**		hypothetical protein; putative non-cytoplasmic protein	CDS	115731	116141	411	1	reverse
**hypothetical protein CDS; putative non-cytoplasmicprotein CDS**		hypothetical protein; putative non-cytoplasmic protein	CDS	112449	112928	480	1	reverse
**hypothetical protein CDS; putative non-cytoplasmicprotein CDS**		hypothetical protein; putative non-cytoplasmic protein	CDS	39172	39483	312	1	reverse
**hypothetical protein CDS; putative non-cytoplasmic protein CDS**		hypothetical protein; putative non-cytoplasmic protein	CDS	8173	8604	432	1	forward
**hypothetical protein CDS; putative non-cytoplasmic protein CDS**		hypothetical protein; putative non-cytoplasmic protein	CDS	3481	4029	549	1	forward
**hypothetical protein CDS; putative non-cytoplasmic protein CDS**		hypothetical protein; putative non-cytoplasmic protein	CDS	3259	3477	219	1	forward
**hypothetical protein CDS; putative non-cytoplasmic protein CDS**		hypothetical protein; putative non-cytoplasmic protein	CDS	2337	3074	738	1	forward
**hypothetical protein CDS; putative non-cytoplasmic protein CDS**		hypothetical protein; putative non-cytoplasmic protein	CDS	141	683	543	1	forward
**hypothetical protein CDS; putative non-cytoplasmic protein CDS**		hypothetical protein; putative non-cytoplasmic protein	CDS	<1	>139031	>534	2	forward
**hypothetical protein CDS; putative nucleotide kinase CDS**		hypothetical protein; putative nucleotide kinase	CDS	135328	135744	417	1	forward
**hypothetical protein CDS; putative transcription regulator CDS**		hypothetical protein; putative transcription regulator	CDS	26920	27036	117	1	reverse
**hypothetical protein CDS; putative virion component CDS**		hypothetical protein; putative virion component	CDS	128129	128743	615	1	forward
**hypothetical protein CDS; putative virion protein CDS**		hypothetical protein; putative virion protein	CDS	113085	113885	801	1	reverse
**hypothetical protein CDS; receptor binding protein CDS**		hypothetical protein; receptor binding protein	CDS	72003	73925	1923	1	reverse
**hypothetical protein CDS; resolvase CDS**		hypothetical protein; resolvase	CDS	58781	59389	609	1	reverse
**hypothetical protein CDS; serine/threonine protein phosphatase CDS**		hypothetical protein; serine/threonine protein phosphatase	CDS	6002	6709	708	1	forward
**hypothetical protein CDS; tail assembly chaperone CDS**		hypothetical protein; tail assembly chaperone	CDS	95154	95612	459	1	reverse
**hypothetical protein CDS; transcriptional regulator CDS**		hypothetical protein; transcriptional regulator	CDS	126623	126853	231	1	forward
**hypothetical protein CDS; transglycosylase CDS**		hypothetical protein; transglycosylase	CDS	1528	1917	390	1	forward
**I-KsaII CDS; ksaII CDS; putative endonuclease CDS**	I-KsaII; ksaII	I-KsaII; KsaII; putative endonuclease	CDS	51785	52297	513	1	reverse
**Ioh CDS; putative group I intron-encoded nuclease CDS**	Ioh	Ioh; putative group I intron-encoded nuclease	CDS	97032	98435	1404	1	reverse
**Iro CDS; iro CDS; putative cytoplasmic protein CDS**	Iro; iro	Iro; putative cytoplasmic protein	CDS	118589	118807	219	1	forward
**RNA ligase CDS; lig CDS**	lig	Lig; RNA ligase	CDS	129828	130724	897	1	forward
**N-acetylmuramoyl-L-alanine amidase CDS; lysK CDS**	lysK	LysK; N-acetylmuramoyl-L-alanine amidase	CDS	121127	123490	1488	2	forward
**mbpA CDS; putative membrane-associated protein CDS**	mbpA	MbpA; putative membrane protein MbpA; putative membrane-associated protein	CDS	8597	9082	486	1	forward
**mbpB CDS; putative membrane-associated protein CDS**	mbpB	MbpB; putative membrane-associated protein	CDS	131336	131914	579	1	forward
**hypothetical protein CDS; mbpC CDS**	mbpC	MbpC; hypothetical protein	CDS	117220	117546	327	1	forward
**mbpD CDS; putative membrane-associated protein CDS**	mbpD	MbpD; putative membrane protein MbpD; putative membrane-associated protein	CDS	116394	116780	387	1	reverse
**mbpE CDS**	mbpE	MbpE; putative membrane protein MbpE	CDS	110806	111156	351	1	reverse
**mbpF CDS; putative membrane-associated protein CDS**	mbpF	MbpF; putative membrane-associated protein; putative membrane protein MbpF	CDS	111232	112407	1176	1	reverse
**hypothetical protein CDS; mbpG CDS**	mbpG	MbpG; hypothetical protein; putative membrane protein MbpG	CDS	39470	39838	369	1	reverse
**mbpH CDS; putative membrane-associated protein CDS**	mbpH	MbpH; putative membrane protein MbpH; putative membrane-associated protein	CDS	33257	33655	399	1	reverse
**mbpI CDS; putative membrane-associated protein CDS**	mbpI	MbpI; putative membrane protein MbpI; putative membrane-associated protein	CDS	29809	30087	279	1	reverse
**mbpJ CDS; putative membrane-associated protein CDS**	mbpJ	MbpJ; putative membrane protein MbpJ; putative membrane-associated protein	CDS	31798	32331	534	1	reverse
**mbpK CDS**	mbpK	MbpK; putative membrane protein MbpK	CDS	30828	31019	192	1	reverse
**mbpP CDS**	mbpP	MbpP; putative membrane protein MbpP	CDS	138472	138636	165	1	forward
**mbpR CDS**	mbpR	MbpR	CDS	138191	138469	279	1	forward
**mbpS CDS; prohibitin domain membrane protein CDS**	mbpS	MbpS; prohibitin domain membrane protein; putative membrane protein MbpS	CDS	123911	124702	792	1	forward
**mbpT CDS; putative membrane-associated protein CDS**	mbpT	MbpT; putative membrane-associated protein	CDS	9079	9270	192	1	forward

**mbpU CDS; putative membrane-associated protein CDS**	mbpU	MbpU; putative membrane-associated protein	CDS	129604	129828	225	1	forward
**mbpV CDS; putative membrane-associated protein CDS**	mbpV	MbpV; putative membrane-associated protein	CDS	123603	123911	309	1	forward
**mbpW CDS; putative membrane-associated protein CDS**	mbpW	MbpW; putative membrane-associated protein	CDS	116921	117187	267	1	forward
**mbpX CDS; putative membrane-associated protein CDS**	mbpX	MbpX; putative membrane-associated protein	CDS	96137	96331	195	1	reverse
**mbpY CDS; putative membrane-associated protein CDS**	mbpY	MbpY; putative membrane-associated protein	CDS	31535	31783	249	1	reverse
**mbpZ CDS**	mbpZ	MbpZ	CDS	26411	26590	180	1	reverse
**major capsid protein CDS; mcp CDS**	mcp	Mcp; major capsid protein	CDS	105279	106670	1392	1	reverse
**nadV CDS**	nadV	NadV	CDS	21567	23036	1470	1	reverse
**nrdF CDS; putative ribonucleotide reductase minor subunit CDS**	nrdF	NrdF; putative ribonucleotide reductase minor subunit	CDS	55180	56229	1050	1	reverse
**nrdI CDS**	nrdI	NrdI	CDS	58372	58803	432	1	reverse
**phosphate starvation-inducible protein CDS; phr CDS**	phr	Phr; phosphate starvation-inducible protein	CDS	128795	129535	741	1	forward
**polA CDS**	polA	PolA	CDS	48237	53335	3219	3	reverse
**polA CDS**	polA	PolA	CDS	48237	53335	3219	3	reverse
**pri CDS; DNA primase CDS**	pri	Pri; DNA primase	CDS	60233	61300	1068	1	reverse
**pro CDS; prohead protease CDS**	pro	Pro; prohead protease	CDS	107755	108528	774	1	reverse
**portal protein CDS; prt CDS**	prt	Prt; portal protein	CDS	108722	110413	1692	1	reverse
**hypothetical protein CDS; pufA CDS**	pufA	PufA; hypothetical protein	CDS	33178	33240	63	1	reverse
**pufB CDS**	pufB	PufB	CDS	28202	28270	69	1	reverse
**pufC CDS**	pufC	PufC	CDS	28064	28138	75	1	reverse
**putative capsid component CDS**		putative capsid component	CDS	42857	42973	117	1	reverse
**putative capsid component CDS**		putative capsid component	CDS	42452	42772	321	1	reverse
**mbpF CDS**	mbpF	putative membrane protein MbpF	CDS	111220	112407	1188	1	reverse
**mbpL CDS; putative membrane-associated protein CDS**	mbpL	putative membrane protein MbpL; putative membrane-associated protein	CDS	27047	27334	288	1	reverse
**mbpM CDS; putative membrane-associated protein CDS**	mbpM	putative membrane protein MbpM; putative membrane-associated protein	CDS	27646	28047	402	1	reverse
**mbpN CDS**	mbpN	putative membrane protein MbpN	CDS	24885	25043	159	1	reverse
**mbpO CDS**	mbpO	putative membrane protein MbpO	CDS	24359	24649	291	1	reverse
**putative membrane-associated protein CDS**		putative membrane-associated protein	CDS	112925	113098	174	1	reverse
**putative membrane-associated protein CDS**		putative membrane-associated protein	CDS	110806	111147	342	1	reverse
**putative membrane-associated protein CDS**		putative membrane-associated protein	CDS	30828	31010	183	1	reverse
**putative non-cytoplasmic protein CDS**		putative non-cytoplasmic protein	CDS	5351	5902	552	1	forward
**putative non-cytoplasmic protein CDS**		putative non-cytoplasmic protein	CDS	1919	2158	240	1	forward
**putative ribonucleotide reductase large subunit CDS; nrdE CDS**	nrdE	putative ribonucleotide reductase large subunit; NrdE	CDS	56243	58357	2115	1	reverse
**rbn CDS; ribonuclease H CDS**	rbn	Rbn; ribonuclease H	CDS	127688	128113	426	1	forward
**rec CDS; recombinase CDS**	rec	Rec; recombinase	CDS	44753	46009	1257	1	reverse
**ribonucleotide reductase stimulatory protein CDS**		ribonucleotide reductase stimulatory protein	CDS	58372	58764	393	1	reverse
**recombination-related endonuclease CDS; rncA CDS**	rncA	RncA; recombination-related endonuclease	CDS	64207	65244	1038	1	reverse
**recombination-related endonuclease CDS; rncB CDS**	rncB	RncB; recombination-related endonuclease	CDS	61911	63830	1920	1	reverse
**rpp CDS**	rpp	Rpp	CDS	23054	23962	909	1	reverse
**hypothetical protein CDS; sci CDS**	sci	Sci; hypothetical protein	CDS	32321	32497	177	1	reverse
**sig CDS; sigma factor CDS**	sig	Sig; sigma factor	CDS	43747	44409	663	1	reverse
**ter CDS; terminase large subunit CDS**	ter	Ter; terminase large subunit	CDS	113899	115716	1818	1	reverse
**lytic transglycosylase CDS; tgl CDS**	tgl	Tgl; lytic transglycosylase	CDS	125591	126283	693	1	forward
**tio CDS**	tio	Tio	CDS	54529	54840	312	1	reverse
**putative oxidoreductase CDS; tio CDS**	tio	Tio; putative oxidoreductase	CDS	54529	54849	321	1	reverse
**tail tube protein CDS; tmpA CDS**	tmpA	TmpA; tail tube protein	CDS	98534	99523	426	3	reverse

**tail tube protein CDS; tmpA CDS**	tmpA	TmpA; tail tube protein	CDS	98534	99523	426	3	reverse
**tmpB CDS**	tmpB	TmpB	CDS	94574	95110	537	1	reverse
**tmpC CDS**	tmpC	TmpC	CDS	90463	94521	4059	1	reverse
**tail murein hydrolase CDS; tmpD CDS**	tmpD	TmpD; tail murein hydrolase	CDS	87958	90384	2427	1	reverse
**putative cysteine protease CDS; tmpE CDS**	tmpE	TmpE; putative cysteine protease	CDS	87057	87944	888	1	reverse
**tmpF CDS**	tmpF	TmpF	CDS	78244	81303	3060	1	reverse
**tmpG CDS**	tmpG	TmpG	CDS	74133	77591	3459	1	reverse
**tmpH CDS**	tmpH	TmpH	CDS	42452	42964	513	1	reverse
**putative capsid component CDS; tail protein CDS; tmpI CDS**	tmpI	TmpI; putative capsid component; tail protein	CDS	42210	42437	228	1	reverse
**treA CDS**	treA	TreA	CDS	17646	17945	300	1	reverse
**treB CDS**	treB	TreB	CDS	17445	17630	186	1	reverse
**treC CDS**	treC	TreC	CDS	17051	17338	288	1	reverse
**treD CDS**	treD	TreD	CDS	16725	17051	327	1	reverse
**treE CDS; hypothetical protein CDS**	treE	TreE; hypothetical protein	CDS	16416	16709	294	1	reverse
**treF CDS**	treF	TreF	CDS	16155	16412	258	1	reverse
**treG CDS**	treG	TreG	CDS	15838	16077	240	1	reverse
**treH CDS**	treH	TreH	CDS	15480	15827	348	1	reverse
**treH CDS**	treH	TreH	CDS	15480	15824	345	1	reverse
**treI CDS**	treI	TreI	CDS	14933	15271	339	1	forward
**treJ CDS; hypothetical protein CDS**	treJ	TreJ; hypothetical protein	CDS	14313	14621	309	1	reverse
**treK CDS**	treK	TreK	CDS	13824	14107	284	1	reverse
**treK CDS**	treK	TreK	CDS	13820	14107	288	1	reverse
**treL CDS**	treL	TreL	CDS	13579	13770	192	1	reverse
**treM CDS**	treM	TreM	CDS	12774	13262	489	1	forward
**treN CDS**	treN	TreN	CDS	12448	12606	159	1	reverse
**treP CDS; hypothetical protein CDS**	treP	TreP; hypothetical protein	CDS	11957	12280	324	1	reverse
**hypothetical protein CDS; treQ CDS**	treQ	TreQ; hypothetical protein	CDS	11621	11857	237	1	reverse
**putative non-cytoplasmic protein CDS; treR CDS**	treR	TreR; putative non-cytoplasmic protein	CDS	11071	11541	471	1	reverse
**treS CDS**	treS	TreS	CDS	10839	11021	183	1	reverse
**hypothetical protein CDS; treS CDS**	treS	TreS; hypothetical protein	CDS	10839	11012	174	1	reverse
**treT CDS; hypothetical protein CDS**	treT	TreT; hypothetical protein	CDS	10570	10839	270	1	reverse
**hypothetical protein CDS; treU CDS**	treU	TreU; hypothetical protein	CDS	10264	10485	222	1	reverse
**integration host factor CDS; trf CDS**	trf	Trf; integration host factor	CDS	53411	53716	306	1	reverse
**tRNA-Asp**		tRNA-Asp	tRNA	119962	120035	74	1	forward
**tRNA-Met**		tRNA-Met	tRNA	4309	4380	72	1	forward
**tRNA-Phe**		tRNA-Phe	tRNA	120042	120114	73	1	forward
**tsp CDS**	tsp	Tsp	CDS	99596	101359	1764	1	reverse
**UboA CDS**		UboA	CDS	9273	9704	432	1	forward
**hypothetical protein CDS; uphA CDS**	uphA	UphA; hypothetical protein	CDS	120354	120539	186	1	forward

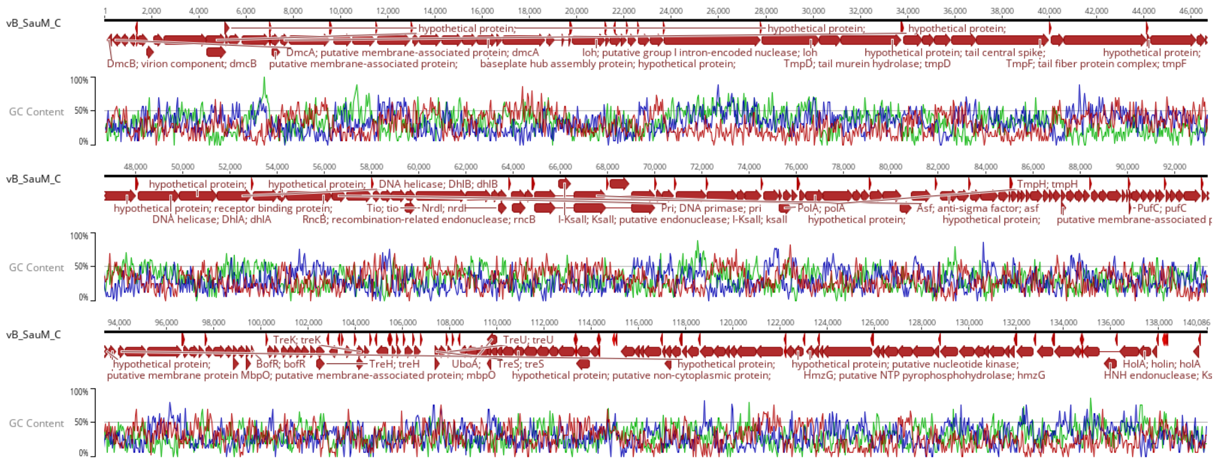

Figure S2. Genome organization of bacteriophage vB_SauM_C

Table S2. Genome annotation of bacteriophage vB_SauM_C

Name	gene	product	Type	Minimum	Maximum	Length	# Intervals	Direction
**hypothetical protein CDS; uphA CDS**	uphA	UphA; hypothetical protein	CDS	137777	137962	186	1	reverse
**UboC CDS; hypothetical protein CDS**		UboC; hypothetical protein	CDS	108679	109164	486	1	reverse
**UboB CDS; hypothetical protein CDS**		UboB; hypothetical protein	CDS	109177	109584	408	1	reverse
**UboA CDS**		UboA	CDS	109584	110015	432	1	reverse
**tsp CDS; tail sheath protein CDS**	tsp	Tsp; tail sheath protein	CDS	16866	18629	1764	1	forward
**tRNA-Phe**		tRNA-Phe	tRNA	138202	138274	73	1	reverse
**tRNA-Met**		tRNA-Met	tRNA	114908	114979	72	1	reverse
**tRNA-Asp**		tRNA-Asp	tRNA	138281	138354	74	1	reverse
**integration host factor CDS; trf CDS**	trf	Trf; integration host factor	CDS	64515	64820	306	1	forward
**treU CDS**	treU	TreU	CDS	107928	108107	180	1	forward
**treT CDS; hypothetical protein CDS**	treT	TreT; hypothetical protein	CDS	107574	107843	270	1	forward
**hypothetical protein CDS; treS CDS**	treS	TreS; hypothetical protein	CDS	107401	107574	174	1	forward
**treS CDS**	treS	TreS	CDS	107392	107574	183	1	forward
**hypothetical protein CDS; treQ CDS**	treQ	TreQ; hypothetical protein	CDS	106520	106756	237	1	forward
**hypothetical protein CDS; treP CDS**	treP	TreP; hypothetical protein	CDS	106097	106420	324	1	forward
**hypothetical protein CDS; treO CDS**	treO	TreO; hypothetical protein	CDS	105798	105929	132	1	forward
**putative membrane-associated protein CDS; treN CDS**	treN	TreN; putative membrane-associated protein	CDS	105570	105728	159	1	forward
**hypothetical protein CDS; treL CDS**	treL	TreL; hypothetical protein	CDS	104406	104597	192	1	forward
**hypothetical protein CDS; treK CDS**	treK	TreK; hypothetical protein	CDS	104069	104356	288	1	forward
**treK CDS**	treK	TreK	CDS	104069	104352	284	1	forward
**hypothetical protein CDS; treJ CDS**	treJ	TreJ; hypothetical protein	CDS	103555	103863	309	1	forward
**hypothetical protein CDS; treI CDS**	treI	TreI; hypothetical protein	CDS	102906	103244	339	1	reverse
**hypothetical protein CDS; treH CDS**	treH	TreH; hypothetical protein	CDS	102352	102699	348	1	forward
**treH CDS**	treH	TreH	CDS	102355	102699	345	1	forward
**treG CDS; hypothetical protein CDS**	treG	TreG; hypothetical protein	CDS	102102	102341	240	1	forward
**hypothetical protein CDS; treF CDS**	treF	TreF; hypothetical protein	CDS	101767	102024	258	1	forward
**treE CDS; hypothetical protein CDS**	treE	TreE; hypothetical protein	CDS	101470	101763	294	1	forward
**hypothetical protein CDS; treD CDS**	treD	TreD; hypothetical protein	CDS	101183	101470	288	1	forward
**hypothetical protein CDS; treC CDS**	treC	TreC; hypothetical protein	CDS	100893	101183	291	1	forward
**putative transmembrane protein CDS; treB CDS**	treB	TreB; putative transmembrane protein	CDS	100601	100786	186	1	forward

**putative non-cytoplasmic protein CDS; treA CDS**	treA	TreA; putative non-cytoplasmic protein	CDS	100286	100585	300	1	forward
**tail protein CDS; tmpI CDS**	tmpI	TmpI; tail protein	CDS	75794	76021	228	1	forward
**tmpH CDS**	tmpH	TmpH	CDS	75267	75779	513	1	forward
**tmpG CDS; adsorption-associated tail protein CDS**	tmpG	TmpG; adsorption-associated tail protein	CDS	40640	44098	3459	1	forward
**tmpF CDS; tail fiber protein complex CDS**	tmpF	TmpF; tail fiber protein complex	CDS	36928	39987	3060	1	forward
**putative cysteine protease CDS; tmpE CDS**	tmpE	TmpE; putative cysteine protease	CDS	30281	31168	888	1	forward
**tail murein hydrolase CDS; tmpD CDS**	tmpD	TmpD; tail murein hydrolase	CDS	27841	30267	2427	1	forward
**tmpC CDS; tape measure protein CDS**	tmpC	TmpC; tape measure protein	CDS	23704	27762	4059	1	forward
**tmpB CDS; tail morphogenetic protein CDS**	tmpB	TmpB; tail morphogenetic protein	CDS	23115	23651	537	1	forward
**tail tube protein CDS; tmpA CDS**	tmpA	TmpA; tail tube protein	CDS	18702	19691	426	3	forward
**putative oxidoreductase CDS; tio CDS**	tio	Tio; putative oxidoreductase	CDS	63382	63702	321	1	forward
**tio CDS**	tio	Tio	CDS	63391	63702	312	1	forward
**lytic transglycosylase CDS; tgl CDS**	tgl	Tgl; lytic transglycosylase	CDS	132033	132725	693	1	reverse
**ter CDS; terminase large subunit CDS**	ter	Ter; terminase large subunit	CDS	2491	4308	1818	1	forward
**sig CDS; sigma factor CDS**	sig	Sig; sigma factor	CDS	73822	74484	663	1	forward
**hypothetical protein CDS; sci CDS**	sci	Sci; hypothetical protein	CDS	85734	85910	177	1	forward
**ribose-phosphate pyrophosphokinase CDS; rpp CDS**	rpp	Rpp; ribose-phosphate pyrophosphokinase	CDS	94269	95177	909	1	forward
**recombination-related endonuclease CDS; rncB CDS**	rncB	RncB; recombination-related endonuclease	CDS	54401	56320	1920	1	forward
**recombination-related endonuclease CDS; rncA CDS**	rncA	RncA; recombination-related endonuclease	CDS	52987	54024	1038	1	forward
**ribonucleotide reductase stimulatory protein CDS**		ribonucleotide reductase stimulatory protein	CDS	59467	59859	393	1	forward
**rec CDS; recombinase CDS**	rec	Rec; recombinase	CDS	72222	73478	1257	1	forward
**rbn CDS; ribonuclease H CDS**	rbn	Rbn; ribonuclease H	CDS	130203	130628	426	1	reverse
**putative virion protein CDS; hypothetical protein CDS**		putative virion protein; hypothetical protein	CDS	4322	5122	801	1	forward
**putative non-cytoplasmic protein CDS; hypothetical protein CDS**		putative non-cytoplasmic protein; hypothetical protein	CDS	119147	119680	534	1	reverse
**putative non-cytoplasmic protein CDS**		putative non-cytoplasmic protein	CDS	117130	117369	240	1	reverse
**putative non-cytoplasmic protein CDS**		putative non-cytoplasmic protein	CDS	113386	113937	552	1	reverse
**putative neck protein CDS; hypothetical protein CDS**		putative neck protein; hypothetical protein	CDS	14269	15147	879	1	forward
**putative membrane-associated protein CDS; mbpP CDS**	mbpP	putative membrane-associated protein; MbpP; putative membrane protein MbpP	CDS	119683	119847	165	1	reverse
**putative membrane-associated protein CDS**		putative membrane-associated protein	CDS	87221	87403	183	1	forward
**putative membrane-associated protein CDS**		putative membrane-associated protein	CDS	7078	7419	342	1	forward
**putative membrane-associated protein CDS**		putative membrane-associated protein	CDS	5109	5282	174	1	forward
**mbpO CDS; putative membrane-associated protein CDS**	mbpO	putative membrane protein MbpO; putative membrane-associated protein	CDS	93582	93872	291	1	forward
**mbpN CDS; putative membrane-associated protein CDS**	mbpN	putative membrane protein MbpN; putative membrane-associated protein	CDS	93188	93346	159	1	forward
**mbpM CDS; putative membrane-associated protein CDS**	mbpM	putative membrane protein MbpM; putative membrane-associated protein	CDS	90184	90585	402	1	forward
**mbpL CDS; putative membrane-associated protein CDS**	mbpL	putative membrane protein MbpL; putative membrane-associated protein	CDS	90897	91184	288	1	forward
**putative capsid component CDS**		putative capsid component	CDS	75459	75779	321	1	forward
**putative capsid component CDS**		putative capsid component	CDS	75258	75374	117	1	forward
**pufC CDS**	pufC	PufC	CDS	90093	90167	75	1	forward
**pufB CDS**	pufB	PufB	CDS	89961	90029	69	1	forward
**hypothetical protein CDS; pufA CDS**	pufA	PufA; hypothetical protein	CDS	84991	85053	63	1	forward
**portal protein CDS; prt CDS**	prt	Prt; portal protein	CDS	7812	9503	1692	1	forward
**pro CDS; prohead protease CDS**	pro	Pro; prohead protease	CDS	9697	10470	774	1	forward
**pri CDS; DNA primase CDS**	pri	Pri; DNA primase	CDS	56931	57998	1068	1	forward

**polA CDS**	polA	PolA	CDS	64896	69994	3219	3	forward
**phosphate starvation-inducible protein CDS; phr CDS**	phr	Phr; phosphate starvation-inducible protein	CDS	128781	129521	741	1	reverse
**nrdI CDS**	nrdI	NrdI	CDS	59428	59859	432	1	forward
**nrdF CDS; putative ribonucleotide reductase minor subunit CDS**	nrdF	NrdF; putative ribonucleotide reductase minor subunit	CDS	62002	63051	1050	1	forward
**nrdE CDS; putative ribonucleotide reductase large subunit CDS**	nrdE	NrdE; putative ribonucleotide reductase large subunit	CDS	59874	61988	2115	1	forward
**nadV CDS; nicotinamide phosphoribosyltransferase CDS**	nadV	NadV; nicotinamide phosphoribosyltransferase	CDS	95195	96664	1470	1	forward
**major capsid protein CDS; mcp CDS**	mcp	Mcp; major capsid protein	CDS	11555	12946	1392	1	forward
**mbpZ CDS; putative membrane-associated protein CDS**	mbpZ	MbpZ; putative membrane-associated protein	CDS	91641	91820	180	1	forward
**mbpY CDS; putative membrane-associated protein CDS**	mbpY	MbpY; putative membrane-associated protein	CDS	86448	86696	249	1	forward
**mbpX CDS; putative membrane-associated protein CDS**	mbpX	MbpX; putative membrane-associated protein	CDS	21894	22088	195	1	forward
**mbpW CDS; putative membrane-associated protein CDS**	mbpW	MbpW; putative membrane-associated protein	CDS	1020	1286	267	1	reverse
**mbpV CDS; putative membrane-associated protein CDS**	mbpV	MbpV; putative membrane-associated protein	CDS	134405	134713	309	1	reverse
**mbpU CDS; putative membrane-associated protein CDS**	mbpU	MbpU; putative membrane-associated protein	CDS	128488	128712	225	1	reverse
**mbpT CDS; putative membrane-associated protein CDS**	mbpT	MbpT; putative membrane-associated protein	CDS	110018	110209	192	1	reverse
**mbpS CDS; prohibitin domain membrane protein CDS**	mbpS	MbpS; prohibitin domain membrane protein; putative membrane protein MbpS	CDS	133614	134405	792	1	reverse
**mbpR CDS; putative membrane-associated protein CDS**	mbpR	MbpR; putative membrane protein MbpR; putative membrane-associated protein	CDS	119850	120125	276	1	reverse
**mbpK CDS**	mbpK	MbpK; putative membrane protein MbpK	CDS	87212	87403	192	1	forward
**mbpJ CDS; putative membrane-associated protein CDS**	mbpJ	MbpJ; putative membrane protein MbpJ; putative membrane-associated protein	CDS	85900	86433	534	1	forward
**mbpI CDS; putative membrane-associated protein CDS**	mbpI	MbpI; putative membrane protein MbpI; putative membrane-associated protein	CDS	88144	88422	279	1	forward
**mbpH CDS; putative membrane-associated protein CDS**	mbpH	MbpH; putative membrane protein MbpH; putative membrane-associated protein	CDS	84576	84974	399	1	forward
**hypothetical protein CDS; mbpG CDS**	mbpG	MbpG; hypothetical protein; putative membrane protein MbpG	CDS	78393	78761	369	1	forward
**mbpF CDS; putative membrane-associated protein CDS**	mbpF	MbpF; putative membrane-associated protein; putative membrane protein MbpF	CDS	5800	6993	1194	1	forward
**mbpE CDS**	mbpE	MbpE; putative membrane protein MbpE	CDS	7069	7419	351	1	forward
**mbpD CDS; putative membrane-associated protein CDS**	mbpD	MbpD; putative membrane protein MbpD; putative membrane-associated protein	CDS	1427	1813	387	1	forward
**hypothetical protein CDS; mbpC CDS**	mbpC	MbpC; hypothetical protein	CDS	661	987	327	1	reverse
**mbpB CDS; putative membrane-associated protein CDS**	mbpB	MbpB; putative membrane-associated protein	CDS	126402	126980	579	1	reverse
**mbpA CDS; putative membrane-associated protein CDS**	mbpA	MbpA; putative membrane protein MbpA; putative membrane-associated protein	CDS	110206	110691	486	1	reverse
**N-acetylmuramoyl-L-alanine amidase CDS; lysK CDS**	lysK	LysK; N-acetylmuramoyl-L-alanine amidase	CDS	134826	137189	1488	2	reverse
**RNA ligase CDS; lig CDS**	lig	Lig; RNA ligase	CDS	127592	128488	897	1	reverse
**Iro CDS; iro CDS; putative cytoplasmic protein CDS**	Iro; iro	Iro; putative cytoplasmic protein	CDS	139509	139727	219	1	reverse
**Ioh CDS; putative group I intron-encoded nuclease CDS**	Ioh	Ioh; putative group I intron-encoded nuclease	CDS	19790	21193	1404	1	forward
**I-KsaII CDS; ksaII CDS; putative endonuclease CDS**	I-KsaII; ksaII	I-KsaII; KsaII; putative endonuclease	CDS	65934	66446	513	1	forward
**hypothetical protein CDS; virion component CDS**		hypothetical protein; virion component	CDS	92419	93099	681	1	forward
**hypothetical protein CDS; transglycosylase CDS**		hypothetical protein; transglycosylase	CDS	117371	117760	390	1	reverse
**hypothetical protein CDS; transcriptional regulator CDS**		hypothetical protein; transcriptional regulator	CDS	131463	131693	231	1	reverse

**hypothetical protein CDS; tail central spike CDS**		hypothetical protein; tail central spike	CDS	31168	33720	2553	1	forward
**hypothetical protein CDS; tail assembly chaperone CDS**		hypothetical protein; tail assembly chaperone	CDS	22613	23071	459	1	forward
**hypothetical protein CDS; serine/threonine protein phosphatase CDS**		hypothetical protein; serine/threonine protein phosphatase	CDS	112579	113286	708	1	reverse
**hypothetical protein CDS; resolvase CDS**		hypothetical protein; resolvase	CDS	58842	59450	609	1	forward
**hypothetical protein CDS; receptor binding protein CDS**		hypothetical protein; receptor binding protein	CDS	46632	48008	1377	1	forward
**hypothetical protein CDS; receptor binding protein CDS**		hypothetical protein; receptor binding protein	CDS	44306	46228	1923	1	forward
**hypothetical protein CDS; putative virion component CDS**		hypothetical protein; putative virion component	CDS	129573	130187	615	1	reverse
**hypothetical protein CDS; putative transcription regulator CDS**		hypothetical protein; putative transcription regulator	CDS	91195	91311	117	1	forward
**hypothetical protein CDS; putative structural protein CDS**		hypothetical protein; putative structural protein	CDS	82827	83285	459	1	forward
**hypothetical protein CDS; putative nucleotide kinase CDS**		hypothetical protein; putative nucleotide kinase	CDS	122572	122988	417	1	reverse
**hypothetical protein CDS; putative non-cytoplasmic protein CDS**		hypothetical protein; putative non-cytoplasmic protein	CDS	130832	131473	642	1	reverse
**hypothetical protein CDS; putative non-cytoplasmic protein CDS**		hypothetical protein; putative non-cytoplasmic protein	CDS	130618	130809	192	1	reverse
**hypothetical protein CDS; putative non-cytoplasmic protein CDS**		hypothetical protein; putative non-cytoplasmic protein	CDS	125942	126205	264	1	reverse
**hypothetical protein CDS; putative non-cytoplasmic protein CDS**		hypothetical protein; putative non-cytoplasmic protein	CDS	123816	125864	2049	1	reverse
**hypothetical protein CDS; putative non-cytoplasmic protein CDS**		hypothetical protein; putative non-cytoplasmic protein	CDS	123655	123816	162	1	reverse
**hypothetical protein CDS; putative non-cytoplasmic protein CDS**		hypothetical protein; putative non-cytoplasmic protein	CDS	123423	123611	189	1	reverse
**hypothetical protein CDS; putative non-cytoplasmic protein CDS**		hypothetical protein; putative non-cytoplasmic protein	CDS	120125	120970	846	1	reverse
**hypothetical protein CDS; putative non-cytoplasmic protein CDS**		hypothetical protein; putative non-cytoplasmic protein	CDS	118605	119147	543	1	reverse
**hypothetical protein CDS; putative non-cytoplasmic protein CDS**		hypothetical protein; putative non-cytoplasmic protein	CDS	116214	116951	738	1	reverse
**hypothetical protein CDS; putative non-cytoplasmic protein CDS**		hypothetical protein; putative non-cytoplasmic protein	CDS	115811	116029	219	1	reverse
**hypothetical protein CDS; putative non-cytoplasmic protein CDS**		hypothetical protein; putative non-cytoplasmic protein	CDS	115259	115807	549	1	reverse
**hypothetical protein CDS; putative non-cytoplasmic protein CDS**		hypothetical protein; putative non-cytoplasmic protein	CDS	110684	111115	432	1	reverse
**hypothetical protein CDS; putative non-cytoplasmic protein CDS**		hypothetical protein; putative non-cytoplasmic protein	CDS	5279	5758	480	1	forward
**hypothetical protein CDS; putative non-cytoplasmic protein CDS**		hypothetical protein; putative non-cytoplasmic protein	CDS	2066	2476	411	1	forward
**hypothetical protein CDS; putative neck protein CDS**		hypothetical protein; putative neck protein	CDS	13347	14255	909	1	forward
**hypothetical protein CDS; putative membrane-associated protein CDS**		hypothetical protein; putative membrane-associated protein	CDS	91301	91564	264	1	forward
**hypothetical protein CDS; putative membrane-associated protein CDS**		hypothetical protein; putative membrane-associated protein	CDS	85519	85698	180	1	forward
**hypothetical protein CDS; putative membrane-associated protein CDS**		hypothetical protein; putative membrane-associated protein	CDS	85368	85532	165	1	forward
**hypothetical protein CDS; putative HNH endonuclease CDS**		hypothetical protein; putative HNH endonuclease	CDS	132912	133547	636	1	reverse
**hypothetical protein CDS; putative endonuclease CDS**		hypothetical protein; putative endonuclease	CDS	89162	89764	603	1	forward
**hypothetical protein CDS; putative DNA repair exonuclease CDS**		hypothetical protein; putative DNA repair exonuclease	CDS	77129	78379	1251	1	forward
**hypothetical protein CDS; putative cytoplasmic protein CDS**		hypothetical protein; putative cytoplasmic protein	CDS	131696	131923	228	1	reverse
**hypothetical protein CDS; putative cytoplasmic protein CDS**		hypothetical protein; putative cytoplasmic protein	CDS	116030	116224	195	1	reverse
**hypothetical protein CDS; putative cytoplasmic protein CDS**		hypothetical protein; putative cytoplasmic protein	CDS	112184	112582	399	1	reverse
**hypothetical protein CDS; putative capsid component CDS**		hypothetical protein; putative capsid component	CDS	74612	75244	633	1	forward
**hypothetical protein CDS; putative baseplate component CDS**		hypothetical protein; putative baseplate component	CDS	46251	46625	375	1	forward

**hypothetical protein CDS; putative baseplate component CDS**		hypothetical protein; putative baseplate component	CDS	34618	35142	525	1	forward
**hypothetical protein CDS; putative baseplate component CDS**		hypothetical protein; putative baseplate component	CDS	33827	34618	792	1	forward
**hypothetical protein CDS; peptidoglycan binding protein CDS**		hypothetical protein; peptidoglycan binding protein	CDS	126222	126395	174	1	reverse
**hypothetical protein CDS; nucleotidyl transferase CDS**		hypothetical protein; nucleotidyl transferase	CDS	111129	111671	543	1	reverse
**hypothetical protein CDS; nucleoside 2-deoxyribosyltransferase CDS**		hypothetical protein; nucleoside 2-deoxyribosyltransferase	CDS	126973	127599	627	1	reverse
**hypothetical protein CDS; major tail protein CDS**		hypothetical protein; major tail protein	CDS	113956	114273	318	1	reverse
**hypothetical protein CDS**		hypothetical protein	CDS	122253	122579	327	1	reverse
**hypothetical protein CDS**		hypothetical protein	CDS	118073	118555	483	1	reverse
**hypothetical protein CDS**		hypothetical protein	CDS	117859	118032	174	1	reverse
**hypothetical protein CDS**		hypothetical protein	CDS	117130	117378	249	1	reverse
**hypothetical protein CDS**		hypothetical protein	CDS	117014	117118	105	1	reverse
**hypothetical protein CDS**		hypothetical protein	CDS	113386	113940	555	1	reverse
**hypothetical protein CDS**		hypothetical protein	CDS	111683	112171	489	1	reverse
**hypothetical protein CDS**		hypothetical protein	CDS	109584	109754	171	1	reverse
**hypothetical protein CDS**		hypothetical protein	CDS	109575	109754	180	1	reverse
**hypothetical protein CDS**		hypothetical protein	CDS	99396	99563	168	1	forward
**hypothetical protein CDS**		hypothetical protein	CDS	99375	99563	189	1	forward
**hypothetical protein CDS**		hypothetical protein	CDS	99067	99381	315	1	forward
**hypothetical protein CDS**		hypothetical protein	CDS	98849	99043	195	1	forward
**hypothetical protein CDS**		hypothetical protein	CDS	98834	99043	210	1	forward
**hypothetical protein CDS**		hypothetical protein	CDS	98514	98843	330	1	forward
**hypothetical protein CDS**		hypothetical protein	CDS	98003	98512	510	1	forward
**hypothetical protein CDS**		hypothetical protein	CDS	97689	98000	312	1	forward
**hypothetical protein CDS**		hypothetical protein	CDS	97402	97623	222	1	forward
**hypothetical protein CDS**		hypothetical protein	CDS	97008	97400	393	1	forward
**hypothetical protein CDS**		hypothetical protein	CDS	96743	96988	246	1	forward
**hypothetical protein CDS**		hypothetical protein	CDS	93964	94272	309	1	forward
**hypothetical protein CDS**		hypothetical protein	CDS	93381	93581	201	1	forward
**hypothetical protein CDS**		hypothetical protein	CDS	92101	92418	318	1	forward
**hypothetical protein CDS**		hypothetical protein	CDS	91835	92098	264	1	forward
**hypothetical protein CDS**		hypothetical protein	CDS	90587	90880	294	1	forward
**hypothetical protein CDS**		hypothetical protein	CDS	90086	90184	99	1	forward
**hypothetical protein CDS**		hypothetical protein	CDS	89778	89957	180	1	forward
**hypothetical protein CDS**		hypothetical protein	CDS	88812	89162	351	1	forward
**hypothetical protein CDS**		hypothetical protein	CDS	88492	88797	306	1	forward
**hypothetical protein CDS**		hypothetical protein	CDS	87797	88144	348	1	forward
**hypothetical protein CDS**		hypothetical protein	CDS	87416	87784	369	1	forward
**hypothetical protein CDS**		hypothetical protein	CDS	86877	87173	297	1	forward
**hypothetical protein CDS**		hypothetical protein	CDS	86708	86884	177	1	forward
**hypothetical protein CDS**		hypothetical protein	CDS	85121	85363	243	1	forward
**hypothetical protein CDS**		hypothetical protein	CDS	83810	84514	705	1	forward
**hypothetical protein CDS**		hypothetical protein	CDS	83350	83793	444	1	forward
**hypothetical protein CDS**		hypothetical protein	CDS	82078	82809	732	1	forward
**hypothetical protein CDS**		hypothetical protein	CDS	80843	81706	864	1	forward
**hypothetical protein CDS**		hypothetical protein	CDS	80397	80843	447	1	forward

**hypothetical protein CDS**		hypothetical protein	CDS	79652	80419	768	1	forward
**hypothetical protein CDS**		hypothetical protein	CDS	79123	79659	537	1	forward
**hypothetical protein CDS**		hypothetical protein	CDS	78748	79059	312	1	forward
**hypothetical protein CDS**		hypothetical protein	CDS	76381	77136	756	1	forward
**hypothetical protein CDS**		hypothetical protein	CDS	76117	76377	261	1	forward
**hypothetical protein CDS**		hypothetical protein	CDS	73482	73835	354	1	forward
**hypothetical protein CDS**		hypothetical protein	CDS	70891	72162	1272	1	forward
**hypothetical protein CDS**		hypothetical protein	CDS	70322	70804	483	1	forward
**hypothetical protein CDS**		hypothetical protein	CDS	70063	70305	243	1	forward
**hypothetical protein CDS**		hypothetical protein	CDS	63945	64505	561	1	forward
**hypothetical protein CDS**		hypothetical protein	CDS	63909	64505	597	1	forward
**hypothetical protein CDS**		hypothetical protein	CDS	63069	63398	330	1	forward
**hypothetical protein CDS**		hypothetical protein	CDS	58403	58855	453	1	forward
**hypothetical protein CDS**		hypothetical protein	CDS	58065	58403	339	1	forward
**hypothetical protein CDS**		hypothetical protein	CDS	54024	54401	378	1	forward
**hypothetical protein CDS**		hypothetical protein	CDS	49860	51473	1614	1	forward
**hypothetical protein CDS**		hypothetical protein	CDS	44147	44305	159	1	forward
**hypothetical protein CDS**		hypothetical protein	CDS	22170	22481	312	1	forward
**hypothetical protein CDS**		hypothetical protein	CDS	21423	21881	459	1	forward
**hypothetical protein CDS**		hypothetical protein	CDS	21240	21380	141	1	forward
**hypothetical protein CDS**		hypothetical protein	CDS	16624	16839	216	1	forward
**hypothetical protein CDS**		hypothetical protein	CDS	15147	15767	621	1	forward
**hypothetical protein CDS**		hypothetical protein	CDS	13038	13334	297	1	forward
**hypothetical protein CDS**		hypothetical protein	CDS	10489	11439	951	1	forward
**hypothetical protein CDS**		hypothetical protein	CDS	7437	7808	372	1	forward
**hypothetical protein CDS**		hypothetical protein	CDS	5100	5282	183	1	forward
**hypothetical protein CDS**		hypothetical protein	CDS	4301	5122	822	1	forward
**holA CDS; holin CDS**	holA	HolA; holin	CDS	137189	137692	504	1	reverse
**HNH homing endonuclease CDS; treM CDS**	treM	HNH homing endonuclease; TreM	CDS	104914	105402	489	1	reverse
**HNH homing endonuclease CDS; I-KsaIII CDS; ksaIII CDS**	I-KsaIII; ksaIII	HNH homing endonuclease; HnhC; KsaIII; I-KsaIII	CDS	68091	68900	810	1	forward
**HNH endonuclease CDS; ksaI CDS**	ksaI	HNH endonuclease; KsaI	CDS	135726	136226	501	1	reverse
**hmzG CDS; putative NTP pyrophosphohydrolase CDS**	hmzG	HmzG; putative NTP pyrophosphohydrolase	CDS	123121	123423	303	1	reverse
**DUF1024 domain protein CDS**		DUF1024 domain protein	CDS	109761	110015	255	1	reverse
**DNA polymerase I CDS**		DNA polymerase I	CDS	64896	69994	3219	3	forward
**DNA helicase CDS; dhlB CDS**	dhlB	DNA helicase; DhlB	CDS	51466	52908	1443	1	forward
**DNA helicase CDS; dhlA CDS**	dhlA	DNA helicase; DhlA	CDS	48100	49848	1749	1	forward
**dmd CDS; putative cytoplasmic protein CDS**	dmd	Dmd; putative cytoplasmic protein	CDS	1791	2069	279	1	forward
**dmcB CDS; virion component CDS**	dmcB	DmcB; virion component	CDS	94	303	210	1	reverse
**dmcA CDS; putative membrane-associated protein CDS**	dmcA	DmcA; putative membrane-associated protein	CDS	316	648	333	1	reverse
**bofR CDS**	bofR	BofR	CDS	99600	99701	102	1	forward
**bofL CDS; putative non-cytoplasmic protein CDS**	bofL	BofL; putative non-cytoplasmic protein	CDS	108441	108677	237	1	reverse
**baseplate protein CDS; bmpC CDS**	bmpC	BmpC; baseplate protein	CDS	40098	40619	522	1	forward
**baseplate component CDS; bmpB CDS**	bmpB	BmpB; baseplate component	CDS	35861	36907	1047	1	forward
**baseplate wedge subunit CDS; bmpA CDS**	bmpA	BmpA; baseplate wedge subunit	CDS	35142	35846	705	1	forward
**baseplate hub assembly protein CDS; hypothetical protein CDS**		baseplate hub assembly protein;	CDS	15786	16622	837	1	forward

		hypothetical protein							
**anti-sigma factor CDS; asf CDS**	asf	Asf; anti-sigma factor	CDS	56320	56916	597	1	forward
**AAA family ATPase CDS; hypothetical protein CDS**		AAA family ATPase; hypothetical protein	CDS	120982	122100	1119	1	reverse

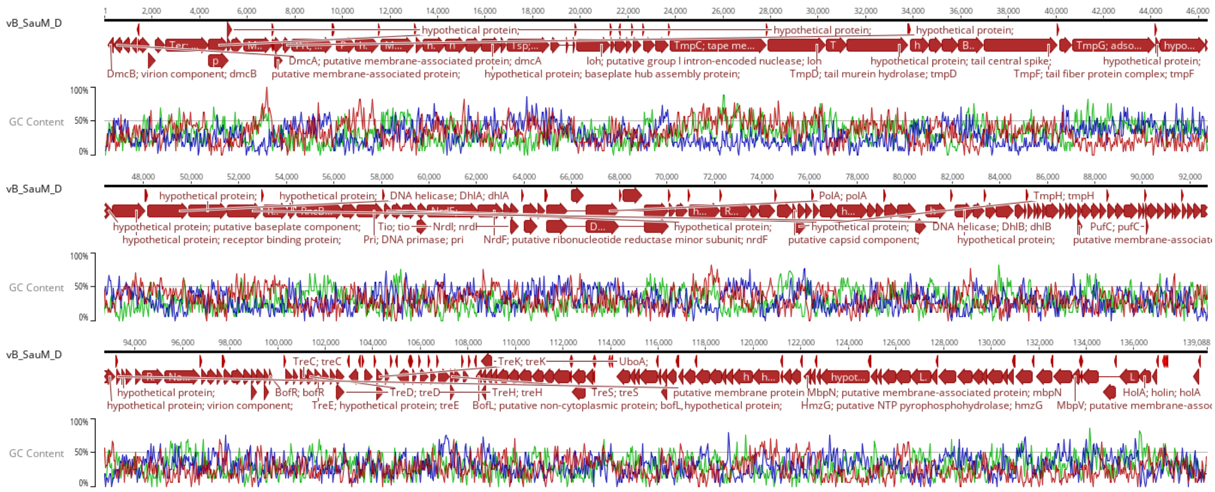

Figure S3. Genome organization of bacteriophage vB_SauM_D

Table S3. Genome annotation of bacteriophage vB_SauM_D

Name	gene	product	Type	Minimum	Maximum	Length	# Intervals	Direction
**hypothetical protein CDS; uphA CDS**	uphA	UphA; hypothetical protein	CDS	136778	136963	186	1	reverse
**UboA CDS**		UboA	CDS	108582	109013	432	1	reverse
**tsp CDS; tail sheath protein CDS**	tsp	Tsp; tail sheath protein	CDS	16922	18685	1764	1	forward
**tRNA-Phe**		tRNA-Phe	tRNA	137203	137275	73	1	reverse
**tRNA-Met**		tRNA-Met	tRNA	113906	113977	72	1	reverse
**tRNA-Asp**		tRNA-Asp	tRNA	137282	137355	74	1	reverse
**integration host factor CDS; trf CDS**	trf	Trf; integration host factor	CDS	64571	64876	306	1	forward
**hypothetical protein CDS; treU CDS**	treU	TreU; hypothetical protein	CDS	107801	108022	222	1	forward
**treT CDS; hypothetical protein CDS**	treT	TreT; hypothetical protein	CDS	107447	107716	270	1	forward
**hypothetical protein CDS; treS CDS**	treS	TreS; hypothetical protein	CDS	107274	107447	174	1	forward
**treS CDS**	treS	TreS	CDS	107265	107447	183	1	forward
**putative non-cytoplasmic protein CDS; treR CDS**	treR	TreR; putative non-cytoplasmic protein	CDS	106745	107215	471	1	forward
**hypothetical protein CDS; treQ CDS**	treQ	TreQ; hypothetical protein	CDS	106429	106665	237	1	forward
**hypothetical protein CDS; treP CDS**	treP	TreP; hypothetical protein	CDS	106006	106329	324	1	forward
**treN CDS; putative membrane-associated protein CDS**	treN	TreN; putative membrane-associated protein	CDS	105680	105838	159	1	forward
**hypothetical protein CDS; treL CDS**	treL	TreL; hypothetical protein	CDS	104516	104707	192	1	forward
**hypothetical protein CDS; treK CDS**	treK	TreK; hypothetical protein	CDS	104179	104466	288	1	forward
**treK CDS**	treK	TreK	CDS	104179	104462	284	1	forward
**treJ CDS; hypothetical protein CDS**	treJ	TreJ; hypothetical protein	CDS	103665	103973	309	1	forward
**hypothetical protein CDS; treI CDS**	treI	TreI; hypothetical protein	CDS	103016	103354	339	1	reverse
**hypothetical protein CDS; treH CDS**	treH	TreH; hypothetical protein	CDS	102460	102807	348	1	forward
**treH CDS**	treH	TreH	CDS	102463	102807	345	1	forward
**treG CDS; hypothetical protein CDS**	treG	TreG; hypothetical protein	CDS	102210	102449	240	1	forward
**hypothetical protein CDS; treF CDS**	treF	TreF; hypothetical protein	CDS	101875	102132	258	1	forward
**treE CDS; hypothetical protein CDS**	treE	TreE; hypothetical protein	CDS	101578	101871	294	1	forward
**treD CDS**	treD	TreD	CDS	101236	101562	327	1	forward
**treC CDS**	treC	TreC	CDS	100949	101236	288	1	forward

**putative transmembrane protein CDS; treB CDS**	treB	TreB; putative transmembrane protein	CDS	100657	100842	186	1	forward
**putative non-cytoplasmic protein CDS; treA CDS**	treA	TreA; putative non-cytoplasmic protein	CDS	100342	100641	300	1	forward
**tail protein CDS; tmpI CDS**	tmpI	TmpI; tail protein	CDS	75850	76077	228	1	forward
**tmpH CDS**	tmpH	TmpH	CDS	75323	75835	513	1	forward
**tmpG CDS; adsorption-associated tail protein CDS**	tmpG	TmpG; adsorption-associated tail protein	CDS	40696	44154	3459	1	forward
**tmpF CDS; tail fiber protein complex CDS**	tmpF	TmpF; tail fiber protein complex	CDS	36984	40043	3060	1	forward
**putative cysteine protease CDS; tmpE CDS**	tmpE	TmpE; putative cysteine protease	CDS	30337	31224	888	1	forward
**tail murein hydrolase CDS; tmpD CDS**	tmpD	TmpD; tail murein hydrolase	CDS	27897	30323	2427	1	forward
**tmpC CDS; tape measure protein CDS**	tmpC	TmpC; tape measure protein	CDS	23760	27818	4059	1	forward
**tmpB CDS; tail morphogenetic protein CDS**	tmpB	TmpB; tail morphogenetic protein	CDS	23171	23707	537	1	forward
**tail tube protein CDS; tmpA CDS**	tmpA	TmpA; tail tube protein	CDS	18758	19747	426	3	forward
**putative oxidoreductase CDS; tio CDS**	tio	Tio; putative oxidoreductase	CDS	63438	63758	321	1	forward
**tio CDS**	tio	Tio	CDS	63447	63758	312	1	forward
**lytic transglycosylase CDS; tgl CDS**	tgl	Tgl; lytic transglycosylase	CDS	131034	131726	693	1	reverse
**ter CDS; terminase large subunit CDS**	ter	Ter; terminase large subunit	CDS	2547	4364	1818	1	forward
**sig CDS; sigma factor CDS**	sig	Sig; sigma factor	CDS	73878	74540	663	1	forward
**hypothetical protein CDS; sci CDS**	sci	Sci; hypothetical protein	CDS	85790	85966	177	1	forward
**ribose-phosphate pyrophosphokinase CDS; rpp CDS**	rpp	Rpp; ribose-phosphate pyrophosphokinase	CDS	94325	95233	909	1	forward
**recombination-related endonuclease CDS; rncB CDS**	rncB	RncB; recombination-related endonuclease	CDS	54457	56376	1920	1	forward
**recombination-related endonuclease CDS; rncA CDS**	rncA	RncA; recombination-related endonuclease	CDS	53043	54080	1038	1	forward
**ribonucleotide reductase stimulatory protein CDS**		ribonucleotide reductase stimulatory protein	CDS	59523	59915	393	1	forward
**rec CDS; recombinase CDS**	rec	Rec; recombinase	CDS	72278	73534	1257	1	forward
**rbn CDS; ribonuclease H CDS**	rbn	Rbn; ribonuclease H	CDS	129204	129629	426	1	reverse
**putative virion protein CDS; hypothetical protein CDS**		putative virion protein; hypothetical protein	CDS	4378	5178	801	1	forward
**putative non-cytoplasmic protein CDS**		putative non-cytoplasmic protein	CDS	116128	116367	240	1	reverse
**putative non-cytoplasmic protein CDS**		putative non-cytoplasmic protein	CDS	112384	112935	552	1	reverse
**putative membrane-associated protein CDS**		putative membrane-associated protein	CDS	87277	87459	183	1	forward
**putative membrane-associated protein CDS**		putative membrane-associated protein	CDS	7134	7475	342	1	forward
**putative membrane-associated protein CDS**		putative membrane-associated protein	CDS	5165	5338	174	1	forward
**mbpO CDS; putative membrane-associated protein CDS**	mbpO	putative membrane protein MbpO; putative membrane-associated protein	CDS	93638	93928	291	1	forward
**mbpN CDS; putative membrane-associated protein CDS**	mbpN	putative membrane protein MbpN; putative membrane-associated protein	CDS	93244	93402	159	1	forward
**mbpM CDS; putative membrane-associated protein CDS**	mbpM	putative membrane protein MbpM; putative membrane-associated protein	CDS	90240	90641	402	1	forward
**mbpL CDS; putative membrane-associated protein CDS**	mbpL	putative membrane protein MbpL; putative membrane-associated protein	CDS	90953	91240	288	1	forward
**putative capsid component CDS**		putative capsid component	CDS	75515	75835	321	1	forward
**putative capsid component CDS**		putative capsid component	CDS	75314	75430	117	1	forward
**pufC CDS**	pufC	PufC	CDS	90149	90223	75	1	forward
**pufB CDS**	pufB	PufB	CDS	90017	90085	69	1	forward
**hypothetical protein CDS; pufA CDS**	pufA	PufA; hypothetical protein	CDS	85047	85109	63	1	forward
**prt CDS; portal protein CDS**	prt	Prt; portal protein	CDS	7868	9559	1692	1	forward
**pro CDS; prohead protease CDS**	pro	Pro; prohead protease	CDS	9753	10526	774	1	forward
**pri CDS; DNA primase CDS**	pri	Pri; DNA primase	CDS	56987	58054	1068	1	forward
**polA CDS**	polA	PolA	CDS	64952	70050	3219	3	forward
**phosphate starvation-inducible protein CDS; phr CDS**	phr	Phr; phosphate starvation-inducible protein	CDS	127782	128522	741	1	reverse

**nrdI CDS**	nrdI	NrdI	CDS	59484	59915	432	1	forward
**nrdF CDS; putative ribonucleotide reductase minor subunit CDS**	nrdF	NrdF; putative ribonucleotide reductase minor subunit	CDS	62058	63107	1050	1	forward
**nrdE CDS; putative ribonucleotide reductase large subunit CDS**	nrdE	NrdE; putative ribonucleotide reductase large subunit	CDS	59930	62044	2115	1	forward
**nadV CDS; nicotinamide phosphoribosyltransferase CDS**	nadV	NadV; nicotinamide phosphoribosyltransferase	CDS	95251	96720	1470	1	forward
**major capsid protein CDS; mcp CDS**	mcp	Mcp; major capsid protein	CDS	11611	13002	1392	1	forward
**mbpZ CDS; putative membrane-associated protein CDS**	mbpZ	MbpZ; putative membrane-associated protein	CDS	91697	91876	180	1	forward
**mbpY CDS; putative membrane-associated protein CDS**	mbpY	MbpY; putative membrane-associated protein	CDS	86504	86752	249	1	forward
**mbpX CDS; putative membrane-associated protein CDS**	mbpX	MbpX; putative membrane-associated protein	CDS	21950	22144	195	1	forward
**mbpW CDS; putative membrane-associated protein CDS**	mbpW	MbpW; putative membrane-associated protein	CDS	1076	1342	267	1	reverse
**mbpV CDS; putative membrane-associated protein CDS**	mbpV	MbpV; putative membrane-associated protein	CDS	133406	133714	309	1	reverse
**mbpU CDS; putative membrane-associated protein CDS**	mbpU	MbpU; putative membrane-associated protein	CDS	127489	127713	225	1	reverse
**mbpT CDS; putative membrane-associated protein CDS**	mbpT	MbpT; putative membrane-associated protein	CDS	109016	109207	192	1	reverse
**mbpS CDS; prohibitin domain membrane protein CDS**	mbpS	MbpS; prohibitin domain membrane protein; putative membrane protein MbpS	CDS	132615	133406	792	1	reverse
**mbpR CDS; putative membrane-associated protein CDS**	mbpR	MbpR; putative membrane protein MbpR; putative membrane-associated protein	CDS	118848	119126	279	1	reverse
**mbpP CDS; putative membrane-associated protein CDS**	mbpP	MbpP; putative membrane protein MbpP; putative membrane-associated protein	CDS	118681	118845	165	1	reverse
**mbpK CDS**	mbpK	MbpK; putative membrane protein MbpK	CDS	87268	87459	192	1	forward
**mbpJ CDS; putative membrane-associated protein CDS**	mbpJ	MbpJ; putative membrane protein MbpJ; putative membrane-associated protein	CDS	85956	86489	534	1	forward
**mbpI CDS; putative membrane-associated protein CDS**	mbpI	MbpI; putative membrane protein MbpI; putative membrane-associated protein	CDS	88200	88478	279	1	forward
**mbpH CDS; putative membrane-associated protein CDS**	mbpH	MbpH; putative membrane protein MbpH; putative membrane-associated protein	CDS	84632	85030	399	1	forward
**hypothetical protein CDS; mbpG CDS**	mbpG	MbpG; hypothetical protein; putative membrane protein MbpG	CDS	78449	78817	369	1	forward
**mbpF CDS; putative membrane-associated protein CDS**	mbpF	MbpF; putative membrane-associated protein; putative membrane protein MbpF	CDS	5856	7049	1194	1	forward
**mbpE CDS**	mbpE	MbpE; putative membrane protein MbpE	CDS	7125	7475	351	1	forward
**mbpD CDS; putative membrane-associated protein CDS**	mbpD	MbpD; putative membrane protein MbpD; putative membrane-associated protein	CDS	1483	1869	387	1	forward
**hypothetical protein CDS; mbpC CDS**	mbpC	MbpC; hypothetical protein	CDS	717	1043	327	1	reverse
**mbpB CDS; putative membrane-associated protein CDS**	mbpB	MbpB; putative membrane-associated protein	CDS	125403	125981	579	1	reverse
**mbpA CDS; putative membrane-associated protein CDS**	mbpA	MbpA; putative membrane protein MbpA; putative membrane-associated protein	CDS	109204	109689	486	1	reverse
**N-acetylmuramoyl-L-alanine amidase CDS; lysK CDS**	lysK	LysK; N-acetylmuramoyl-L-alanine amidase	CDS	133827	136190	1488	2	reverse
**RNA ligase CDS; lig CDS**	lig	Lig; RNA ligase	CDS	126593	127489	897	1	reverse
**Iro CDS; iro CDS; putative cytoplasmic protein CDS**	Iro; iro	Iro; putative cytoplasmic protein	CDS	138510	138728	219	1	reverse
**Ioh CDS; putative group I intron-encoded nuclease CDS**	Ioh	Ioh; putative group I intron-encoded nuclease	CDS	19846	21249	1404	1	forward
**I-KsaII CDS; ksaII CDS; putative endonuclease CDS**	I-KsaII; ksaII	I-KsaII; KsaII; putative endonuclease	CDS	65990	66502	513	1	forward
**hypothetical protein CDS; virion component CDS**		hypothetical protein; virion component	CDS	92475	93155	681	1	forward
**hypothetical protein CDS; transglycosylase CDS**		hypothetical protein; transglycosylase	CDS	116369	116758	390	1	reverse
**hypothetical protein CDS; transcriptional regulator CDS**		hypothetical protein; transcriptional regulator	CDS	130464	130694	231	1	reverse
**hypothetical protein CDS; tail central spike CDS**		hypothetical protein; tail central spike	CDS	31224	33776	2553	1	forward
**hypothetical protein CDS; tail assembly chaperone CDS**		hypothetical protein; tail assembly chaperone	CDS	22669	23127	459	1	forward
**hypothetical protein CDS; serine/threonine protein phosphatase CDS**		hypothetical protein; serine/threonine protein phosphatase	CDS	111577	112284	708	1	reverse

**hypothetical protein CDS; resolvase CDS**		hypothetical protein; resolvase	CDS	58898	59506	609	1	forward
**hypothetical protein CDS; receptor binding protein CDS**		hypothetical protein; receptor binding protein	CDS	46688	48064	1377	1	forward
**hypothetical protein CDS; receptor binding protein CDS**		hypothetical protein; receptor binding protein	CDS	44362	46284	1923	1	forward
**hypothetical protein CDS; putative virion component CDS**		hypothetical protein; putative virion component	CDS	128574	129188	615	1	reverse
**hypothetical protein CDS; putative transcription regulator CDS**		hypothetical protein; putative transcription regulator	CDS	91251	91367	117	1	forward
**hypothetical protein CDS; putative structural protein CDS**		hypothetical protein; putative structural protein	CDS	82883	83341	459	1	forward
**hypothetical protein CDS; putative nucleotide kinase CDS**		hypothetical protein; putative nucleotide kinase	CDS	121573	121989	417	1	reverse
**hypothetical protein CDS; putative non-cytoplasmic protein CDS**		hypothetical protein; putative non-cytoplasmic protein	CDS	129833	130474	642	1	reverse
**hypothetical protein CDS; putative non-cytoplasmic protein CDS**		hypothetical protein; putative non-cytoplasmic protein	CDS	129619	129810	192	1	reverse
**hypothetical protein CDS; putative non-cytoplasmic protein CDS**		hypothetical protein; putative non-cytoplasmic protein	CDS	124943	125206	264	1	reverse
**hypothetical protein CDS; putative non-cytoplasmic protein CDS**		hypothetical protein; putative non-cytoplasmic protein	CDS	122817	124865	2049	1	reverse
**hypothetical protein CDS; putative non-cytoplasmic protein CDS**		hypothetical protein; putative non-cytoplasmic protein	CDS	122656	122817	162	1	reverse
**hypothetical protein CDS; putative non-cytoplasmic protein CDS**		hypothetical protein; putative non-cytoplasmic protein	CDS	122424	122612	189	1	reverse
**hypothetical protein CDS; putative non-cytoplasmic protein CDS**		hypothetical protein; putative non-cytoplasmic protein	CDS	119126	119971	846	1	reverse
**hypothetical protein CDS; putative non-cytoplasmic protein CDS**		hypothetical protein; putative non-cytoplasmic protein	CDS	118145	118678	534	1	reverse
**hypothetical protein CDS; putative non-cytoplasmic protein CDS**		hypothetical protein; putative non-cytoplasmic protein	CDS	117603	118145	543	1	reverse
**hypothetical protein CDS; putative non-cytoplasmic protein CDS**		hypothetical protein; putative non-cytoplasmic protein	CDS	115212	115949	738	1	reverse
**hypothetical protein CDS; putative non-cytoplasmic protein CDS**		hypothetical protein; putative non-cytoplasmic protein	CDS	114809	115027	219	1	reverse
**hypothetical protein CDS; putative non-cytoplasmic protein CDS**		hypothetical protein; putative non-cytoplasmic protein	CDS	114257	114805	549	1	reverse
**hypothetical protein CDS; putative non-cytoplasmic protein CDS**		hypothetical protein; putative non-cytoplasmic protein	CDS	109682	110113	432	1	reverse
**hypothetical protein CDS; putative non-cytoplasmic protein CDS**		hypothetical protein; putative non-cytoplasmic protein	CDS	5335	5814	480	1	forward
**hypothetical protein CDS; putative non-cytoplasmic protein CDS**		hypothetical protein; putative non-cytoplasmic protein	CDS	2122	2532	411	1	forward
**hypothetical protein CDS; putative neck protein CDS**		hypothetical protein; putative neck protein	CDS	14325	15203	879	1	forward
**hypothetical protein CDS; putative neck protein CDS**		hypothetical protein; putative neck protein	CDS	13403	14311	909	1	forward
**hypothetical protein CDS; putative membrane-associated protein CDS**		hypothetical protein; putative membrane-associated protein	CDS	91357	91620	264	1	forward
**hypothetical protein CDS; putative membrane-associated protein CDS**		hypothetical protein; putative membrane-associated protein	CDS	85575	85754	180	1	forward
**hypothetical protein CDS; putative membrane-associated protein CDS**		hypothetical protein; putative membrane-associated protein	CDS	85424	85588	165	1	forward
**hypothetical protein CDS; putative HNH endonuclease CDS**		hypothetical protein; putative HNH endonuclease	CDS	131913	132548	636	1	reverse
**hypothetical protein CDS; putative endonuclease CDS**		hypothetical protein; putative endonuclease	CDS	89218	89820	603	1	forward
**hypothetical protein CDS; putative DNA repair exonuclease CDS**		hypothetical protein; putative DNA repair exonuclease	CDS	77185	78435	1251	1	forward
**hypothetical protein CDS; putative cytoplasmic protein CDS**		hypothetical protein; putative cytoplasmic protein	CDS	130697	130924	228	1	reverse
**hypothetical protein CDS; putative cytoplasmic protein CDS**		hypothetical protein; putative cytoplasmic protein	CDS	115028	115222	195	1	reverse
**hypothetical protein CDS; putative cytoplasmic protein CDS**		hypothetical protein; putative cytoplasmic protein	CDS	111182	111580	399	1	reverse
**hypothetical protein CDS; putative capsid component CDS**		hypothetical protein; putative capsid component	CDS	74668	75300	633	1	forward
**hypothetical protein CDS; putative baseplate component CDS**		hypothetical protein; putative baseplate component	CDS	46307	46681	375	1	forward
**hypothetical protein CDS; putative baseplate component CDS**		hypothetical protein; putative baseplate component	CDS	34674	35198	525	1	forward

**hypothetical protein CDS; putative baseplate component CDS**		hypothetical protein; putative baseplate component	CDS	33883	34674	792	1	forward
**hypothetical protein CDS; peptidoglycan binding protein CDS**		hypothetical protein; peptidoglycan binding protein	CDS	125223	125396	174	1	reverse
**hypothetical protein CDS; nucleotidyl transferase CDS**		hypothetical protein; nucleotidyl transferase	CDS	110127	110669	543	1	reverse
**hypothetical protein CDS; nucleoside 2-deoxyribosyltransferase CDS**		hypothetical protein; nucleoside 2-deoxyribosyltransferase	CDS	125974	126600	627	1	reverse
**hypothetical protein CDS; major tail protein CDS**		hypothetical protein; major tail protein	CDS	112954	113271	318	1	reverse
**hypothetical protein CDS; baseplate hub assembly protein CDS**		hypothetical protein; baseplate hub assembly protein	CDS	15842	16678	837	1	forward
**hypothetical protein CDS; AAA family ATPase CDS**		hypothetical protein; AAA family ATPase	CDS	119983	121101	1119	1	reverse
**hypothetical protein CDS**		hypothetical protein	CDS	121254	121580	327	1	reverse
**hypothetical protein CDS**		hypothetical protein	CDS	117071	117553	483	1	reverse
**hypothetical protein CDS**		hypothetical protein	CDS	116857	117030	174	1	reverse
**hypothetical protein CDS**		hypothetical protein	CDS	116128	116376	249	1	reverse
**hypothetical protein CDS**		hypothetical protein	CDS	116012	116116	105	1	reverse
**hypothetical protein CDS**		hypothetical protein	CDS	112384	112938	555	1	reverse
**hypothetical protein CDS**		hypothetical protein	CDS	110681	111169	489	1	reverse
**hypothetical protein CDS**		hypothetical protein	CDS	108582	108752	171	1	reverse
**hypothetical protein CDS**		hypothetical protein	CDS	108573	108752	180	1	reverse
**hypothetical protein CDS**		hypothetical protein	CDS	99452	99619	168	1	forward
**hypothetical protein CDS**		hypothetical protein	CDS	99431	99619	189	1	forward
**hypothetical protein CDS**		hypothetical protein	CDS	99123	99437	315	1	forward
**hypothetical protein CDS**		hypothetical protein	CDS	98905	99099	195	1	forward
**hypothetical protein CDS**		hypothetical protein	CDS	98890	99099	210	1	forward
**hypothetical protein CDS**		hypothetical protein	CDS	98570	98899	330	1	forward
**hypothetical protein CDS**		hypothetical protein	CDS	98059	98568	510	1	forward
**hypothetical protein CDS**		hypothetical protein	CDS	97745	98056	312	1	forward
**hypothetical protein CDS**		hypothetical protein	CDS	97458	97679	222	1	forward
**hypothetical protein CDS**		hypothetical protein	CDS	97064	97456	393	1	forward
**hypothetical protein CDS**		hypothetical protein	CDS	96799	97044	246	1	forward
**hypothetical protein CDS**		hypothetical protein	CDS	94020	94328	309	1	forward
**hypothetical protein CDS**		hypothetical protein	CDS	93437	93637	201	1	forward
**hypothetical protein CDS**		hypothetical protein	CDS	92157	92474	318	1	forward
**hypothetical protein CDS**		hypothetical protein	CDS	91891	92154	264	1	forward
**hypothetical protein CDS**		hypothetical protein	CDS	90643	90936	294	1	forward
**hypothetical protein CDS**		hypothetical protein	CDS	90142	90240	99	1	forward
**hypothetical protein CDS**		hypothetical protein	CDS	89834	90013	180	1	forward
**hypothetical protein CDS**		hypothetical protein	CDS	88868	89218	351	1	forward
**hypothetical protein CDS**		hypothetical protein	CDS	88548	88853	306	1	forward
**hypothetical protein CDS**		hypothetical protein	CDS	87853	88200	348	1	forward
**hypothetical protein CDS**		hypothetical protein	CDS	87472	87840	369	1	forward
**hypothetical protein CDS**		hypothetical protein	CDS	86933	87229	297	1	forward
**hypothetical protein CDS**		hypothetical protein	CDS	86764	86940	177	1	forward
**hypothetical protein CDS**		hypothetical protein	CDS	85177	85419	243	1	forward
**hypothetical protein CDS**		hypothetical protein	CDS	83866	84570	705	1	forward
**hypothetical protein CDS**		hypothetical protein	CDS	83406	83849	444	1	forward
**hypothetical protein CDS**		hypothetical protein	CDS	82134	82865	732	1	forward

**hypothetical protein CDS**		hypothetical protein	CDS	80899	81762	864	1	forward
**hypothetical protein CDS**		hypothetical protein	CDS	80453	80899	447	1	forward
**hypothetical protein CDS**		hypothetical protein	CDS	79708	80475	768	1	forward
**hypothetical protein CDS**		hypothetical protein	CDS	79179	79715	537	1	forward
**hypothetical protein CDS**		hypothetical protein	CDS	78804	79115	312	1	forward
**hypothetical protein CDS**		hypothetical protein	CDS	76437	77192	756	1	forward
**hypothetical protein CDS**		hypothetical protein	CDS	76173	76433	261	1	forward
**hypothetical protein CDS**		hypothetical protein	CDS	73538	73891	354	1	forward
**hypothetical protein CDS**		hypothetical protein	CDS	70947	72218	1272	1	forward
**hypothetical protein CDS**		hypothetical protein	CDS	70378	70860	483	1	forward
**hypothetical protein CDS**		hypothetical protein	CDS	70119	70361	243	1	forward
**hypothetical protein CDS**		hypothetical protein	CDS	64001	64561	561	1	forward
**hypothetical protein CDS**		hypothetical protein	CDS	63965	64561	597	1	forward
**hypothetical protein CDS**		hypothetical protein	CDS	63125	63454	330	1	forward
**hypothetical protein CDS**		hypothetical protein	CDS	58459	58911	453	1	forward
**hypothetical protein CDS**		hypothetical protein	CDS	58121	58459	339	1	forward
**hypothetical protein CDS**		hypothetical protein	CDS	54080	54457	378	1	forward
**hypothetical protein CDS**		hypothetical protein	CDS	49916	51529	1614	1	forward
**hypothetical protein CDS**		hypothetical protein	CDS	44203	44361	159	1	forward
**hypothetical protein CDS**		hypothetical protein	CDS	22226	22537	312	1	forward
**hypothetical protein CDS**		hypothetical protein	CDS	21479	21937	459	1	forward
**hypothetical protein CDS**		hypothetical protein	CDS	21296	21436	141	1	forward
**hypothetical protein CDS**		hypothetical protein	CDS	16680	16895	216	1	forward
**hypothetical protein CDS**		hypothetical protein	CDS	15203	15823	621	1	forward
**hypothetical protein CDS**		hypothetical protein	CDS	13094	13390	297	1	forward
**hypothetical protein CDS**		hypothetical protein	CDS	10545	11495	951	1	forward
**hypothetical protein CDS**		hypothetical protein	CDS	7493	7864	372	1	forward
**hypothetical protein CDS**		hypothetical protein	CDS	5156	5338	183	1	forward
**hypothetical protein CDS**		hypothetical protein	CDS	4357	5178	822	1	forward
**holA CDS; holin CDS**	holA	HolA; holin	CDS	136190	136693	504	1	reverse
**HNH homing endonuclease CDS; treM CDS**	treM	HNH homing endonuclease; TreM	CDS	105024	105512	489	1	reverse
**HNH homing endonuclease CDS; I-KsaIII CDS; ksaIII CDS**	I-KsaIII; ksaIII	HNH homing endonuclease; HnhC; KsaIII; I-KsaIII	CDS	68147	68956	810	1	forward
**HNH endonuclease CDS; ksaI CDS**	ksaI	HNH endonuclease; KsaI	CDS	134727	135227	501	1	reverse
**hmzG CDS; putative NTP pyrophosphohydrolase CDS**	hmzG	HmzG; putative NTP pyrophosphohydrolase	CDS	122122	122424	303	1	reverse
**DUF1024 domain protein CDS**		DUF1024 domain protein	CDS	108759	109013	255	1	reverse
**DNA polymerase I CDS**		DNA polymerase I	CDS	64952	70050	3219	3	forward
**DNA helicase CDS; dhlB CDS**	dhlB	DNA helicase; DhlB	CDS	51522	52964	1443	1	forward
**DNA helicase CDS; dhlA CDS**	dhlA	DNA helicase; DhlA	CDS	48156	49904	1749	1	forward
**dmd CDS; putative cytoplasmic protein CDS**	dmd	Dmd; putative cytoplasmic protein	CDS	1847	2125	279	1	forward
**dmcB CDS; virion component CDS**	dmcB	DmcB; virion component	CDS	150	359	210	1	reverse
**dmcA CDS; putative membrane-associated protein CDS**	dmcA	DmcA; putative membrane-associated protein	CDS	372	704	333	1	reverse
**bofR CDS**	bofR	BofR	CDS	99656	99757	102	1	forward
**bofL CDS; putative non-cytoplasmic protein CDS**	bofL	BofL; putative non-cytoplasmic protein	CDS	108346	108582	237	1	reverse
**baseplate protein CDS; bmpC CDS**	bmpC	BmpC; baseplate protein	CDS	40154	40675	522	1	forward

**baseplate component CDS; bmpB CDS**	bmpB	BmpB; baseplate component	CDS	35917	36963	1047	1	forward
**baseplate wedge subunit CDS; bmpA CDS**	bmpA	BmpA; baseplate wedge subunit	CDS	35198	35902	705	1	forward
**anti-sigma factor CDS; asf CDS**	asf	Asf; anti-sigma factor	CDS	56376	56972	597	1	forward
